# Machine learning approaches for prediction of early death among lung cancer patients with bone metastases using routine clinical characteristics: An analysis of 19,887 patients

**DOI:** 10.3389/fpubh.2022.1019168

**Published:** 2022-10-06

**Authors:** Yunpeng Cui, Xuedong Shi, Shengjie Wang, Yong Qin, Bailin Wang, Xiaotong Che, Mingxing Lei

**Affiliations:** ^1^Department of Orthopedic Surgery, Peking University First Hospital, Beijing, China; ^2^Department of Orthopaedic Surgery, Shanghai Sixth People's Hospital Affiliated to Shanghai Jiao Tong University, Shanghai, China; ^3^Department of Joint and Sports Medicine Surgery, The Second Affiliated Hospital of Harbin Medical University, Harbin, China; ^4^Department of Thoracic Surgery, Hainan Hospital of Chinese PLA General Hospital, Sanya, China; ^5^Department of Evaluation Office, Hainan Cancer Hospital, Haikou, China; ^6^Department of Orthopedic Surgery, Hainan Hospital of PLA General Hospital, Sanya, China; ^7^Chinese PLA Medical School, Beijing, China; ^8^National Clinical Research Center for Orthopedics, Sports Medicine and Rehabilitation, Chinese PLA General Hospital, Beijing, China

**Keywords:** lung cancer, bone metastasis, machine learning, mortality, clinical decision-making, model explainability

## Abstract

**Purpose:**

Bone is one of the most common sites for the spread of malignant tumors. Patients with bone metastases whose prognosis was shorter than 3 months (early death) were considered as surgical contraindications. However, the information currently available in the literature limits our capacity to assess the risk likelihood of 3 month mortality. As a result, the study's objective is to create an accurate prediction model utilizing machine-learning techniques to predict 3 month mortality specifically among lung cancer patients with bone metastases according to easily available clinical data.

**Methods:**

This study enrolled 19,887 lung cancer patients with bone metastases between 2010 and 2018 from a large oncologic database in the United States. According to a ratio of 8:2, the entire patient cohort was randomly assigned to a training (*n* = 15881, 80%) and validation (*n* = 4,006, 20%) group. In the training group, prediction models were trained and optimized using six approaches, including logistic regression, XGBoosting machine, random forest, neural network, gradient boosting machine, and decision tree. There were 13 metrics, including the Brier score, calibration slope, intercept-in-large, area under the curve (AUC), and sensitivity, used to assess the model's prediction performance in the validation group. In each metric, the best prediction effectiveness was assigned six points, while the worst was given one point. The model with the highest sum score of the 13 measures was optimal. The model's explainability was performed using the local interpretable model-agnostic explanation (LIME) according to the optimal model. Predictor importance was assessed using H_2_O automatic machine learning. Risk stratification was also evaluated based on the optimal threshold.

**Results:**

Among all recruited patients, the 3 month mortality was 48.5%. Twelve variables, including age, primary site, histology, race, sex, tumor (T) stage, node (N) stage, brain metastasis, liver metastasis, cancer-directed surgery, radiation, and chemotherapy, were significantly associated with 3 month mortality based on multivariate analysis, and these variables were included for developing prediction models. With the highest sum score of all the measurements, the gradient boosting machine approach outperformed all the other models (62 points), followed by the XGBooting machine approach (59 points) and logistic regression (53). The area under the curve (AUC) was 0.820 (95% confident interval [CI]: 0.807–0.833), 0.820 (95% CI: 0.807–0.833), and 0.815 (95% CI: 0.801–0.828), respectively, calibration slope was 0.97, 0.95, and 0.96, respectively, and accuracy was all 0.772. Explainability of models was conducted to rank the predictors and visualize their contributions to an individual's mortality outcome. The top four important predictors in the population according to H_2_O automatic machine learning were chemotherapy, followed by liver metastasis, radiation, and brain metastasis. Compared to patients in the low-risk group, patients in the high-risk group were more than three times the odds of dying within 3 months (*P* < 0.001).

**Conclusions:**

Using machine learning techniques, this study offers a number of models, and the optimal model is found after thoroughly assessing and contrasting the prediction performance of each model. The optimal model can be a pragmatic risk prediction tool and is capable of identifying lung cancer patients with bone metastases who are at high risk for 3 month mortality, informing risk counseling, and aiding clinical treatment decision-making. It is better advised for patients in the high-risk group to have radiotherapy alone, the best supportive care, or minimally invasive procedures like cementoplasty.

## Introduction

Lung cancer is a major public health concern worldwide. Its mortality came in first and its incidence came in second among all cancer survivors. In 2020, 2.2 million new lung cancer cases were diagnosed, making for 11.4% of all new cases, and in the same year ~1.8 million deaths were reported based on the Global Cancer Statistics (1). Additionally, the number of lung cancer patients was continuing to increase and was estimated to be doubled in 2040 (1).

Metastasis in lung cancer is a multiple process involving several isolated and overlapping steps such as angiogenesis, hypoxia, circulation, and establishment of a metastatic cancer focus (2, 3). Bone is one of the most typical places for lung cancer patients to develop metastases (4). Lung cancer cells and bone-forming cells interact in two directions, which gives malignant cancers a selection advantage and allows them to both destroy bone and produce new bone (4). It was estimated that bone metastases, primarily in the form of osteolytic disease, occurred in 13.2% (5) to 53% (6) of lung cancer patients. Bone metastases could lead to skeletal-related events, which remarkably affected the quality of life and shortened the survival time of lung cancer patients by half (7, 8).

Lung cancer patients who had bone metastases had a somewhat poor prognosis for survival, with a median survival period of roughly 4.0 to 5.0 months (9, 10). The goal of treatment for this disease was to preserve or enhance the patient's quality of remaining life. As new treatment modalities such as targeted therapy, new agents, and immunotherapy improved (11, 12), surgical treatments to assist reduce skeletal-related problems also increased (13). To explain, secure long-bone fixation and decompressive spine surgery were able to maintain or enhance ambulatory function and overall performance status, but surgery was also a two-edged weapon since improper surgical interventions might accelerate patients' death.

In such a situation, determining the best therapeutic strategy became a major concern, requiring doctors to strike a balance between the risk associated with cancer therapies and quality of life for the remaining life period among those patients. Survival estimation seemed to be a critical step and was able to guide the management of bone metastases. Broadly speaking, it was no longer advised to perform surgery on patients with a life expectancy of <3 months (14–16). Consequently, accurate survival prediction is crucial since over- or underestimating the likelihood of survival might give rise to ineffective treatment, such as either overly aggressive or insufficient therapies.

Machine learning is a collection of approaches that involve exploring non-linear associations between inputs and outputs, and further assessing the probability of the outcome in another dataset using developed algorithms (17, 18). Machine learning was widely and increasingly used in early cancer detection, treatments, and the molecular processes that underlie cancer (17, 19, 20). Notably, machine learning outperformed conventional eligibility criteria (20) and other non-machine learning methods (21).

The study's objective is to develop a reliable prediction model for predicting 3 month mortality specifically among lung cancer patients with bone metastases. Six approaches including logistic regression, XGBoosting machine, random forest, neural network, gradient boosting machine, and decision tree were introduced. There were 13 metrics used to assess and compare the performance of models' predictions. To accurately predict survival prognosis among those patients, this study hypothesized that machine learning approaches were able to develop a series of models and the optimal model could be found via comparisons.

## Methods

### Patients and study design

The study analyzed 61,036 patients with bone metastases between 2010 and 2018 from a large oncologic database in the United States (https://seer.cancer.gov/). This database, which receives funding from the Surveillance Research Program at the National Cancer Institute, serves as an authoritative source of information on the country's 28.0% cancer prevalence rate. The database can be accessed publicly and provide patient data without specific identification, so ethics approval and informed consent were not required. We obtained approval to access the database of the National Cancer Institute in the United States using the reference number (23489-Nov2020). The human data was in accordance with the Helsinki Declaration.

**Figure 1:**
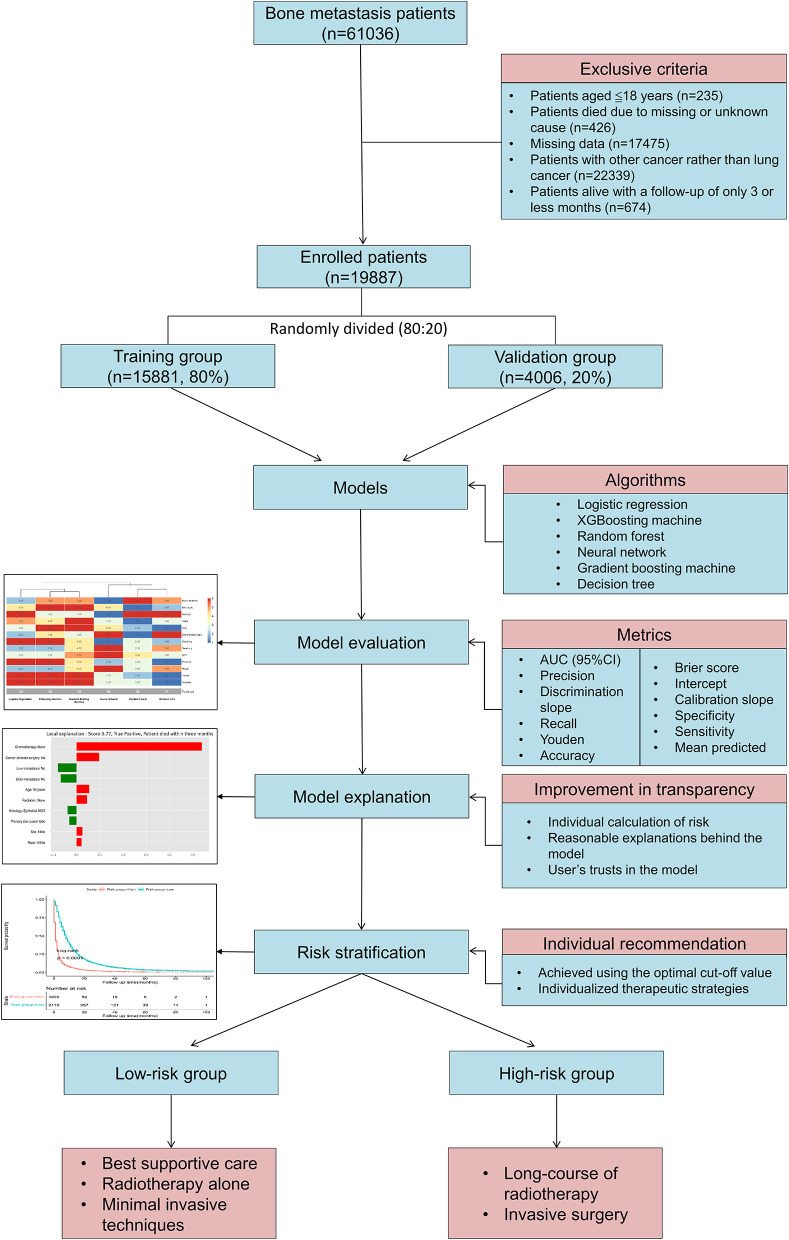
Patient's flowchart and study design.

Patients with lung cancer and bone metastases were included for analysis. The following were the exclusive criteria: (1) Patients had to be 18 years of age or older; (2) Patients died due to missing or undetermined reasons; (3) Missing data; (4) Patients had cancer other than lung cancer; (5) Patient were alive with a follow-up of <3 months. Patients with bone metastases who met the inclusive and exclusive criteria were enrolled (*n* = 19,887). A diagram of patients is shown in Figure 1. All patients were randomly split 8:2 into a training group (*n* = 15,881, 80%) and a validation group (*n* = 4,006, 20%). Patients in the training group were used to train and optimize models, and patients in the validation group were used to validate models.

### Potential predictors

There were 13 variables from the database included for analysis, including age, primary site [main bronchus vs. upper lobe vs. middle lobe vs. lower lobe vs. overlapping lesion vs. lung, not otherwise specified (NOS)], histology (unspecified neoplasms vs. epithelial neoplasms (NOS) vs. squamous cell neoplasms vs. adenomas and adenocarcinomas vs. others), race (black vs. others vs. unknown vs. white), sex (female vs. male), Tumor (T) stage (T0 vs. T1 vs. T2 vs. T3 vs. T4 vs. TX), Node (N) stage (N0 vs. N1 vs. N2 vs. N3 vs. NX), brain metastasis (no vs. unknown vs. yes), liver metastasis (no vs. unknown vs. yes), lung metastasis (no vs. unknown vs. yes), cancer-directed surgery (no vs. unknown vs. yes), radiation (none/unknown vs. yes), and chemotherapy (none/unknown vs. yes). The above variables were common clinical data that were easily available. Based on the American Joint Committee on Cancer (AJCC) and Extent of Disease (EOD) classification, the tumor stage and node stage were recorded. Patients who underwent surgery for any cancer site, including metastasis resulting from primary cancer, were considered to have cancer-directed surgery. We summarized a table outlining information on cancer-directed surgery (Supplementary Table 1). The term “Radiation” refers to the type of radiation therapy used as part of the initial course of treatment. It is a recode of North American Association of Central Cancer Registries (NAACCR) Item (Supplementary Table 2). Chemotherapy records whether chemotherapy was given (Supplementary Table 3). Early death was defined as patients who died within 3 months. Patients having a follow-up of <3 months who were still alive were excluded from the analysis. The health screening characteristics, such as body mass index, radiographic information, and comorbidities were not available in the database.

### Model development

Significant variables were incorporated into the model predictors after multivariate analysis. In the training group, prediction models were trained and optimized using six approaches, including logistic regression, XGBoosting machine, random forest, neural network, gradient boosting machine, and decision tree. The optimal model parameters were found using grid-search and random hyper-parameter search. The models were trained to employ 5-fold cross-validation and 100 iterations of bootstrapping procedures.

### Model selection

The models' prediction performance was evaluated in the validation group using 13 measures, including mean predicted probability of 3 month mortality, the Brier score, intercept-in-large, calibration slope, area under curve (AUC), discrimination slope, specificity, sensitivity, negative predictive value (NPV), PPV/precision, recall, Youden, and accuracy. In all approaches, a threshold was also calculated. The best prediction effectiveness was assigned six points and the worst prediction effectiveness was assigned 1 point in each metric in order to comprehensively compare the prediction performance of the six approaches and determine the optimal one. The optimal model was the approach with the highest sum score of the 13 measures. The heatmap was used to visualize the data using the R “pheatmap” package. Decision curve analysis was employed to calculate the net benefits in a range of threshold probabilities in order to determine the therapeutic applicability of models.

### Model explainability

The local interpretable model-agnostic explanation (LIME) in the optimal model was used to assess the model's explainability. LIME can explain individual prediction of classifier and is able to interpret the output to comprehend how predictions vary with changing observations after adding weights to input (22). As a result, not only can outcome prediction boost user trust in the prediction model, but also plausible justifications and high transparency.

### Importance of model predictors

In the training and validation groups, the importance of predictors was assessed using H_2_O automatic machine learning (Shapley Additive exPlanations, SHAP). H_2_O is an open-source, in-memory, distributed, fast, and scalable machine learning and predictive analytics platform that allows users to build machine learning models on big data and provides easy productionalization of those models (https://docs.h2o.ai/h2o/latest-stable/h2o-docs/index.html).

### Risk stratification

To individually perform therapeutic interventions, risk stratification was achieved based on the optimal cut-off value (the average threshold of the six algorithms). Patients with risk probabilities of below the optimal cut-off value were categorized into the low-risk category, while patients with risk probabilities above the value were categorized into the high-risk category. The study also calculated the matching actual and anticipated probabilities.

### Statistical analysis

Category variables were shown as proportions, while continuous variables were shown as mean and standard deviation (SD). The distribution of categorical variables was compared using Chi-square test and adjusted continuity Chi-square test, while the distribution of continuous variables was compared using *t*-test. Subgroup analysis was employed to analyze patients with and without cancer-directed surgery. Significant variables linked to 3 month mortality were identified using multivariate analysis. The survival curves for the 11 significant categorical variables were plotted by the R “survival” and “survminer” packages, and the log-rank test was used to compare difference in each variable. The relationship between age and the outcome was also investigated using H_2_O automatic machine learning. R programming language version 4.1.2 (https://www.r-project.org/) was used for data visualizations and statistical analysis, while Python version 3.9.7 was used for machine learning processes and model explainability. All codes were available at https://github.com/Starxueshu/Code-and-Model. A *P*-value of < 0.05 was considered statistically significant, and all *P*-values were two-tailed.

## Results

### Patient's characteristics

A total of 19,887 patients with a mean age of 68.46 (SD: 11.05) years were included in the analysis (Table 1). Among all enrolled patients, the majority of primary sites was the upper lobe (48.0%), followed by the lower lobe (24.6%), and the most common histology was adenomas and adenocarcinomas (51.1%), and epithelia neoplasm (NOS) ranked the second (30.1%). Those patients seemed in an advanced stage because more than half of patients had T3 or T4 stage and above 65.0% of patients had N2 or N3 stage. in addition to bone metastases, the percentages of brain, liver, and lung metastases were 23.4, 30.5, and 25.7%, respectively. In terms of cancer therapy, the vast majority of patients (98.8%) did not receive cancer-directed surgery, and more than half received radiation or chemotherapy. The median time to death was 4.00 (95% CI: 3.88–4.12) months. The 3 month mortality was up to 48.5%, indicating near half the patients were dead within 3 months.

**Table 1:** Patient's demographics, clinical characteristics, and therapeutic strategies.

**Characteristics**	**Overall**	**Training group**	**Validation group**	***P* ^a^**
*n*	19,887	15,881	4,006	
Age [mean (SD)]	68.46 (11.05)	68.44 (11.06)	68.53 (11.00)	0.674
Primary site (%)				0.457
Main bronchus	1,001 (5.0)	810 (5.1)	191 (4.8)	
Upper lobe	9,538 (48.0)	7,560 (47.6)	1,978 (49.4)	
Middle lobe	784 (3.9)	624 (3.9)	160 (4.0)	
Lower lobe	4,888 (24.6)	3,928 (24.7)	960 (24.0)	
Overlapping lesion	195 (1.0)	159 (1.0)	36 (0.9)	
Lung, NOS	3,481 (17.5)	2,800 (17.6)	681 (17.0)	
Histology (%)				0.157
Unspecified neoplasms	760 (3.8)	596 (3.8)	164 (4.1)	
Epithelial neoplasms, NOS	5,985 (30.1)	4,753 (29.9)	1,232 (30.8)	
Squamous cell neoplasms	2,444 (12.3)	1,952 (12.3)	492 (12.3)	
Adenomas and adenocarcinomas	10,155 (51.1)	8,126 (51.2)	2,029 (50.6)	
Others	543 (2.7)	454 (2.9)	89 (2.2)	
Race (%)				0.364
Black	2,287 (11.5)	1,805 (11.4)	482 (12.0)	
Others^b^	1,873 (9.4)	1,517 (9.6)	356 (8.9)	
Unknown	21 (0.1)	18 (0.1)	3 (0.1)	
White	15,706 (79.0)	12,541 (79.0)	3,165 (79.0)	
Sex (%)				0.515
Female	8,756 (44.0)	7,011 (44.1)	1,745 (43.6)	
Male	11,131 (56.0)	8,870 (55.9)	2,261 (56.4)	
T stage (%)				0.659
T0	159 (0.8)	130 (0.8)	29 (0.7)	
T1	1,979 (10.0)	1,557 (9.8)	422 (10.5)	
T2	4,313 (21.7)	3,435 (21.6)	878 (21.9)	
T3	4,361 (21.9)	3,508 (22.1)	853 (21.3)	
T4	6,037 (30.4)	4,817 (30.3)	1,220 (30.5)	
TX	3,038 (15.3)	2,434 (15.3)	604 (15.1)	
N stage (%)				0.909
N0	3,686 (18.5)	2,936 (18.5)	750 (18.7)	
N1	1,595 (8.0)	1,267 (8.0)	328 (8.2)	
N2	8,667 (43.6)	6,941 (43.7)	1,726 (43.1)	
N3	4,509 (22.7)	3,605 (22.7)	904 (22.6)	
NX	1,430 (7.2)	1,132 (7.1)	298 (7.4)	
Brain metastasis (%)				0.144
No	14,599 (73.4)	11,654 (73.4)	2,945 (73.5)	
Unknown	635 (3.2)	489 (3.1)	146 (3.6)	
Yes	4,653 (23.4)	3,738 (23.5)	915 (22.8)	
Liver metastasis (%)				0.299
No	13,240 (66.6)	10,572 (66.6)	2,668 (66.6)	
Unknown	589 (3.0)	456 (2.9)	133 (3.3)	
Yes	6,058 (30.5)	4,853 (30.6)	1,205 (30.1)	
Lung metastasis (%)				0.074
No	13,880 (69.8)	11,143 (70.2)	2,737 (68.3)	
Unknown	901 (4.5)	713 (4.5)	188 (4.7)	
Yes	5,106 (25.7)	4,025 (25.3)	1,081 (27.0)	
Cancer-directed surgery (%)				0.284
No	19,645 (98.8)	15,679 (98.7)	3,966 (99.0)	
Unknown	12 (0.1)	11 (0.1)	1 (0.0)	
Yes	230 (1.2)	191 (1.2)	39 (1.0)	
Radiation (%)				0.791
None/unknown	9,859 (49.6)	7,881 (49.6)	1,978 (49.4)	
Yes	10,028 (50.4)	8,000 (50.4)	2,028 (50.6)	
Chemotherapy (%)				0.311
None/unknown	9,035 (45.4)	7,186 (45.2)	1,849 (46.2)	
Yes	10,852 (54.6)	8,695 (54.8)	2,157 (53.8)	
Three month mortality				0.132
No	10,232 (51.5)	8,214 (51.7)	2,018 (50.4)	
Yes	9,655 (48.5)	7,667 (48.3)	1,988 (49.6)	

^a^Indicates continuity adjusted Chi-Square; ^b^Indicates American Indian/AK Native, Asian/Pacific Islander.

SD, standard deviation; NOS, not otherwise specified; T stage, tumor stage; N stage, node stage.

Subgroup analysis was employed to analyze patients with and without cancer-directed surgery, and it demonstrated that patients with cancer-directed surgery tended to be younger (64.05 vs. 68.51 years, *P* < 0.001), and have more adenomas and adenocarcinoma histology (61.7% vs. 50.9%, *P* < 0.001), earlier T (*P* < 0.001) and N (*P* < 0.001) stages, fewer metastases (*P* < 0.001), and more chemotherapy (60.4% vs. 54.5%, *P* = 0.001). Therefore, the early mortality of patients who underwent cancer-directed surgery was significantly lower than that of patients without cancer-directed surgery (24.8% vs. 48.8%, *P* < 0.001). More information is shown in Supplementary Table 4.

### Model development

Patients were separated into two groups at random, and a comparison between the two groups was done (Table 1). In the training group, these variables were all similarly distributed, as shown by the *P*-values that were all more than 0.05. The 13 variables were all significantly different between patients with and without 3 month mortality (Table 2). Furthermore, 12 variables, including age, primary site, histology, race, sex, T and N stage, brain metastasis, liver metastasis, cancer-directed surgery, radiation, and chemotherapy, were also found to be significantly associated with the 3 month mortality (Table 2) and were included for development of prediction models. Six approaches, including logistic regression, XGBoosting machine, random forest, neural network, gradient boosting machine, and decision tree, were conducted to train and optimize prediction models. Supplementary Table 5 provides an overview of the full parameter weights of all techniques.

**Table 2:** Univariate and multivariate analysis of risk factors for 3 month mortality among bone metastasis patients with lung cancer in the training group.

**Characteristics**	**Overall**	**Three month mortality**		***P* ^a^**	**Multivariate analysis**	
		**No**	**No**		**OR (95% CI)**	***P***
*n*	15,881	8,214	7,667		1.66 (0.91–3.04)	0.100
Age [mean (SD)]	68.44 (11.06)	66.43 (10.87)	70.60 (10.86)	<0.001	1.01 (1.01–1.02)	<0.001
Primary site (%)				<0.001		
Main bronchus	810 (5.1)	393 (4.8)	417 (5.4)		Reference	
Upper lobe	7,560 (47.6)	4,076 (49.6)	3,484 (45.4)		0.80 (0.67–0.96)	0.014
Middle lobe	624 (3.9)	352 (4.3)	272 (3.5)		0.77 (0.59–1.00)	0.047
Lower lobe	3,928 (24.7)	2,105 (25.6)	1,823 (23.8)		0.80 (0.66–0.96)	0.018
Overlapping lesion	159 (1.0)	90 (1.1)	69 (0.9)		0.84 (0.55–1.28)	0.418
Lung, NOS	2,800 (17.6)	1,198 (14.6)	1,602 (20.9)		1.07 (0.87–1.30)	0.524
Histology (%)				<0.001		
Unspecified neoplasms	596 (3.8)	95 (1.2)	501 (6.5)		Reference	
Epithelial neoplasms, NOS	4,753 (29.9)	2,337 (28.5)	2,416 (31.5)		0.77 (0.60–0.99)	0.038
Squamous cell neoplasms	1,952 (12.3)	873 (10.6)	1,079 (14.1)		0.83 (0.64–1.09)	0.180
Adenomas and adenocarcinomas	8,126 (51.2)	4,665 (56.8)	3,461 (45.1)		0.63 (0.49–0.80)	<0.001
Others	454 (2.9)	244 (3.0)	210 (2.7)		0.78 (0.56–1.08)	0.135
Race (%)				<0.001		
Black	1,805 (11.4)	921 (11.2)	884 (11.5)		Reference	
Others^b^	1,517 (9.6)	933 (11.4)	584 (7.6)		0.82 (0.68–0.97)	0.021
Unknown	18 (0.1)	9 (0.1)	9 (0.1)		1.08 (0.35–3.33)	0.892
White	12,541 (79.0)	6,351 (77.3)	6,190 (80.7)		1.20 (1.06–1.36)	0.005
Sex (%)				<0.001		
Female	7,011 (44.1)	3,836 (46.7)	3,175 (41.4)		Reference	
Male	8,870 (55.9)	4,378 (53.3)	4,492 (58.6)		1.34 (1.24–1.45)	<0.001
T stage (%)				<0.001		
T0	130 (0.8)	62 (0.8)	68 (0.9)		Reference	
T1	1,557 (9.8)	949 (11.6)	608 (7.9)		0.63 (0.40–0.98)	0.041
T2	3,435 (21.6)	1,861 (22.7)	1,574 (20.5)		0.79 (0.51–1.22)	0.289
T3	3,508 (22.1)	1,768 (21.5)	1,740 (22.7)		0.88 (0.57–1.37)	0.578
T4	4,817 (30.3)	2,515 (30.6)	2,302 (30.0)		0.83 (0.54–1.28)	0.407
TX	2,434 (15.3)	1,059 (12.9)	1,375 (17.9)		0.89 (0.57–1.37)	0.584
N stage (%)				<0.001		
N0	2,936 (18.5)	1,583 (19.3)	1,353 (17.6)		Reference	
N1	1,267 (8.0)	681 (8.3)	586 (7.6)		1.21 (1.03–1.43)	0.024
N2	6,941 (43.7)	3,553 (43.3)	3,388 (44.2)		1.52 (1.36–1.69)	<0.001
N3	3,605 (22.7)	1,969 (24.0)	1,636 (21.3)		1.55 (1.37–1.76)	<0.001
NX	1,132 (7.1)	428 (5.2)	704 (9.2)		1.42 (1.18–1.71)	<0.001
Brain metastasis (%)				<0.001		
No	11,654 (73.4)	6,174 (75.2)	5,480 (71.5)		Reference	
Unknown	489 (3.1)	185 (2.3)	304 (4.0)		1.05 (0.80–1.37)	0.745
Yes	3,738 (23.5)	1,855 (22.6)	1,883 (24.6)		1.58 (1.43–1.75)	<0.001
Liver metastasis (%)				<0.001		
No	10,572 (66.6)	5,883 (71.6)	4,689 (61.2)		Reference	
Unknown	456 (2.9)	202 (2.5)	254 (3.3)		1.06 (0.81–1.40)	0.669
Yes	4,853 (30.6)	2,129 (25.9)	2,724 (35.5)		1.73 (1.58–1.88)	<0.001
Lung metastasis (%)				<0.001		
No	11,143 (70.2)	5,958 (72.5)	5,185 (67.6)		Reference	
Unknown	713 (4.5)	330 (4.0)	383 (5.0)		0.83 (0.67–1.03)	0.098
Yes	4,025 (25.3)	1,926 (23.4)	2,099 (27.4)		1.10 (1.00–1.21)	0.061
Cancer-directed surgery (%)				<0.001		
No	15,679 (98.7)	8,062 (98.1)	7,617 (99.3)		Reference	
Unknown	11 (0.1)	5 (0.1)	6 (0.1)		0.32 (0.08–1.23)	0.097
Yes	191 (1.2)	147 (1.8)	44 (0.6)		0.33 (0.22–0.50)	<0.001
Radiation (%)				<0.001		
None/unknown	7,881 (49.6)	3,422 (41.7)	4,459 (58.2)		Reference	
Yes	8,000 (50.4)	4,792 (58.3)	3,208 (41.8)		0.74 (0.68–0.80)	<0.001
Chemotherapy (%)				<0.001		
None/unknown	7,186 (45.2)	1,480 (18.0)	5,706 (74.4)		Reference	
Yes	8,695 (54.8)	6,734 (82.0)	1,961 (25.6)		0.08 (0.07–0.08)	<0.001

^a^Indicates continuity adjusted Chi-Square; ^b^Indicates American Indian/AK Native, Asian/Pacific Islander.

OR, odds ratio; CI, confident interval; SD, standard deviation; NOS, not otherwise specified; T stage, tumor stage; N stage, node stage.

### Model selection

**Figure 2:**
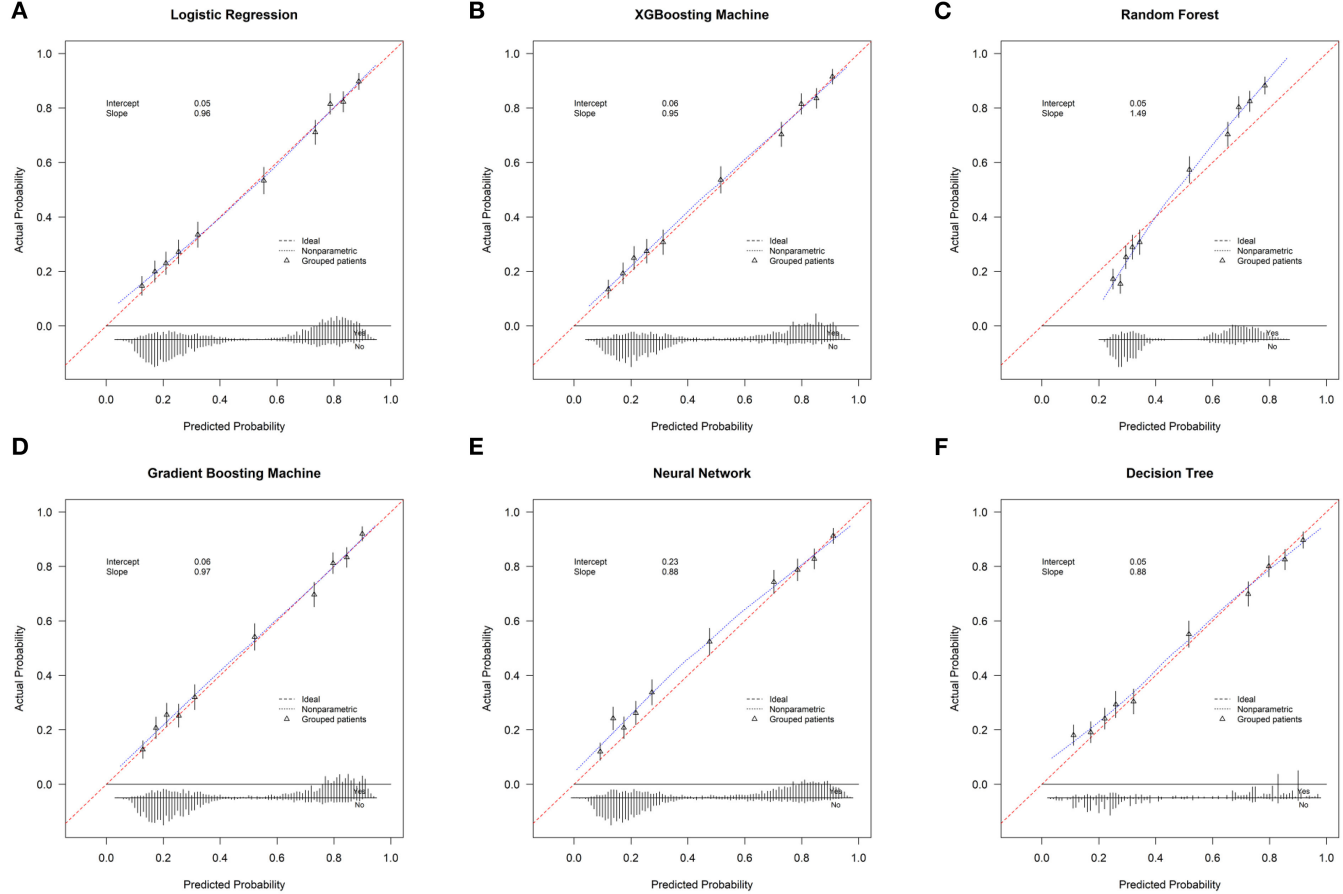
The calibration curves. **(A)** Logistic Regression; **(B)** XGBooting Machine; **(C)** Random Forest; **(D)** Gradient Boosting Machine; **(E)** Neural Network; **(F)** Decision Tree.

**Figure 3:**
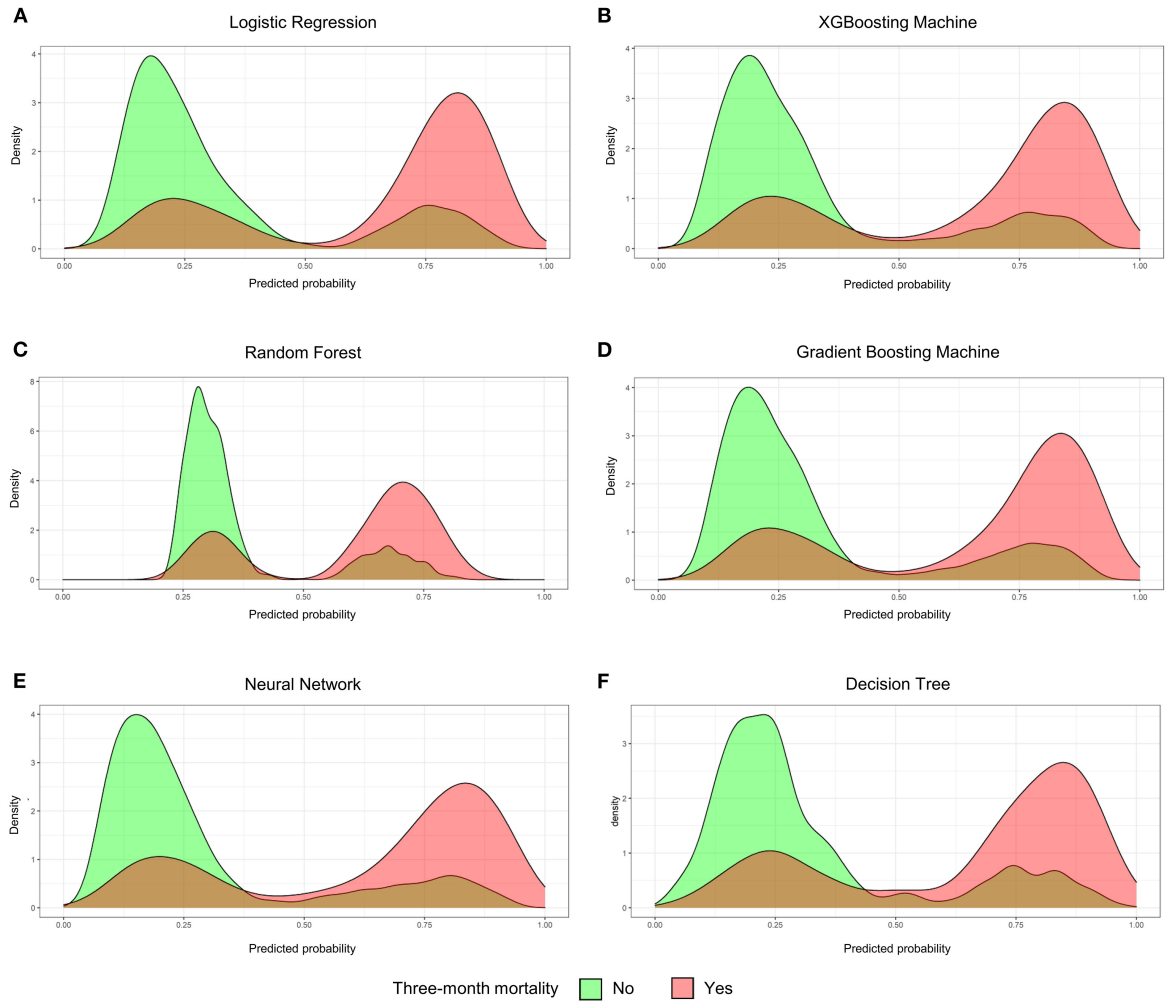
The probability curves. **(A)** Logistic Regression; **(B)** XGBooting Machine; **(C)** Random Forest; **(D)** Gradient Boosting Machine; **(E)** Neural Network; **(F)** Decision Tree. The green group indicates patients were alive for above 3 months and the red group indicates patients were died at or within 3 months.

**Figure 4:**
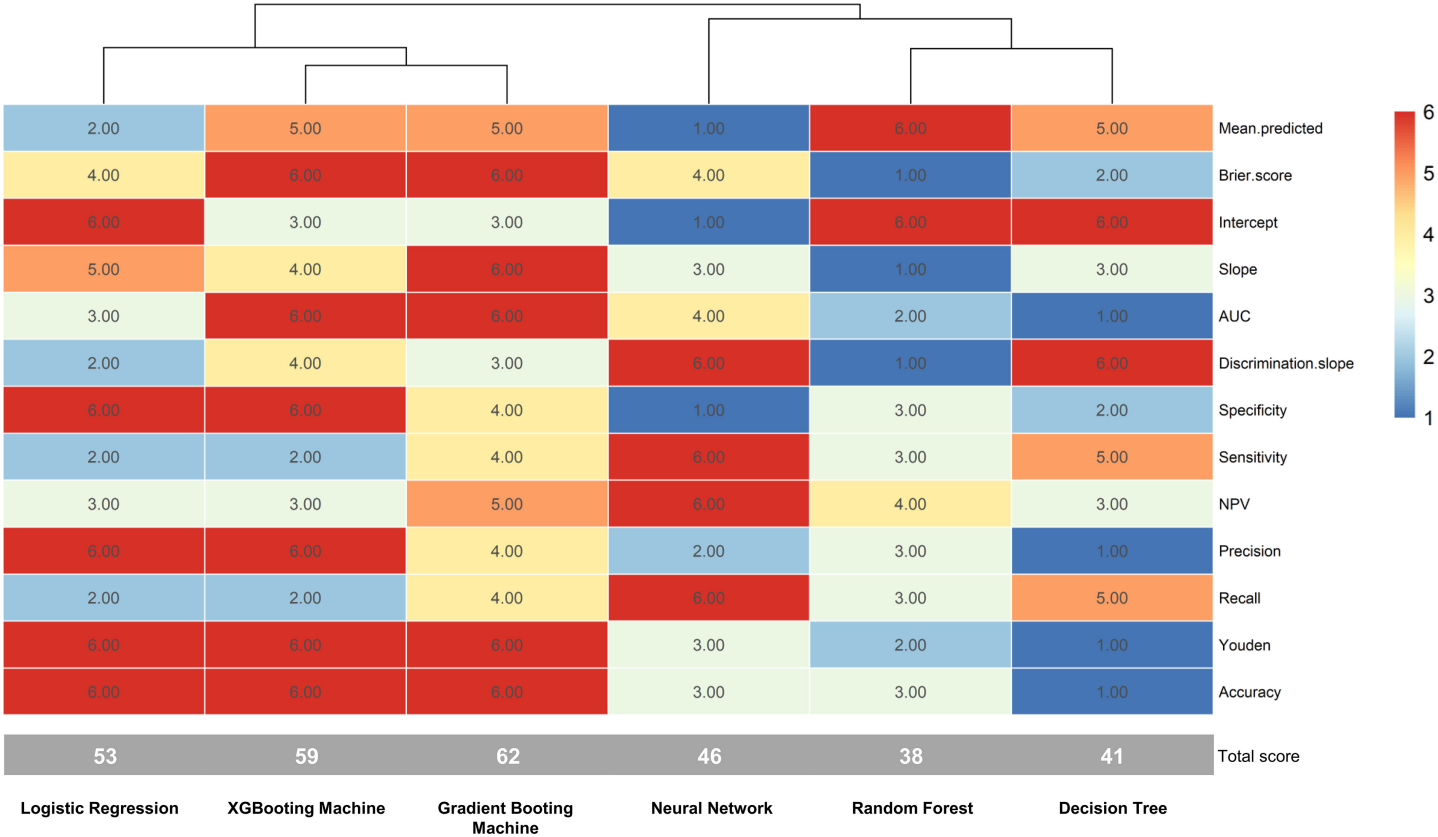
The heatmap of prediction metrics for the six techniques. AUC, area under curve; NPV, negative predictive value.

The validation group used 13 measures to assess models' prediction performance. The AUC for the gradient boosting machine and XGBoosting machine was both 0.820 (95% confident interval [CI]: 0.807–0.833), for the neural network approach it was 0.818 (95% CI: 0.805–0.832), for the logistic regression model it was 0.815 (95% CI: 0.801–0.828), for the random forest approach it was 0.811 (95% CI: 0.798–0.824), and for the decision tree approach it was 0.806 (0.792–0.820) (Supplementary Figure 1). The corresponding calibration slopes were 0.97, 0.95, 0.88, 0.96, 1.49, and 0.88, respectively (Figure 2), and the discrimination slopes were 0.334, 0.338, 0.349, 0.327, 0.228, and 0.349, respectively (Supplementary Figure 2). More information about the prediction measures is summarized in Table 3. Probability curves were plotted for each approach (Figure 3). With the least overlap in probability curves and the greatest separation of the two groups, these models—in particular the gradient boosting machine, XGBoosting machine, and logistic regression—demonstrated significant separation of the curves. To comprehensively evaluate the predictive performance and finally find out the optimal one, the optimal prediction effectiveness was assigned six points and the worst prediction effectiveness was assigned one point in each measure. The heatmap for prediction effectiveness among the six approaches is displayed in Figure 4. The gradient boosting machine approach performed best with the highest sum score of all the measures (62 points), followed by the XGBooting machine approach (59 points) and logistic regression (53 points). Additionally, decision curve analysis revealed that all approaches, especially the gradient boosting machine, had favorable clinical usefulness (Supplementary Figure 3). As a result, the model developed by the gradient boosting machine approach was used to present model explainability.

**Table 3:** Prediction performance of machine learning algorithms for the estimation of 3 month mortality among bone metastasis patients with lung cancer.

**Measures**	**Approaches**					
	**Logistic regression**	**XGBoosting machine**	**Random forest**	**Gradient boosting machine**	**Neural network**	**Decision tree**
Mean predicted	0.488	0.487	0.486	0.487	0.461	0.487
Brier score	0.171	0.169	0.178	0.169	0.171	0.175
Intercept	0.05	0.06	0.05	0.06	0.23	0.05
Slope	0.96	0.95	1.49	0.97	0.88	0.88
AUC (95%CI)	0.815 (0.801–0.828)	0.820 (0.807–0.833)	0.811 (0.798–0.824)	0.820 (0.807–0.833)	0.818 (0.805–0.832)	0.806 (0.792–0.820)
Discrimination slope	0.327	0.338	0.228	0.334	0.349	0.349
Specificity	0.812	0.812	0.807	0.809	0.799	0.800
Sensitivity	0.731	0.731	0.733	0.734	0.742	0.735
NPV	0.754	0.754	0.755	0.756	0.759	0.754
PPV	0.793	0.793	0.789	0.791	0.785	0.784
Precision	0.793	0.793	0.789	0.791	0.785	0.784
Recall	0.731	0.731	0.733	0.734	0.742	0.735
Youden	1.543	1.543	1.541	1.543	1.542	1.536
Accuracy	0.772	0.772	0.771	0.772	0.771	0.768
Threshold	0.526	0.488	0.558	0.466	0.382	0.444

AUC, Are under the curve; CI, Confident interval; NPV, Negative predictive value; PPV, Positive predictive value; XGBooting, eXtreme Gradient Boosting.

### Model's explainability

**Figure 5:**
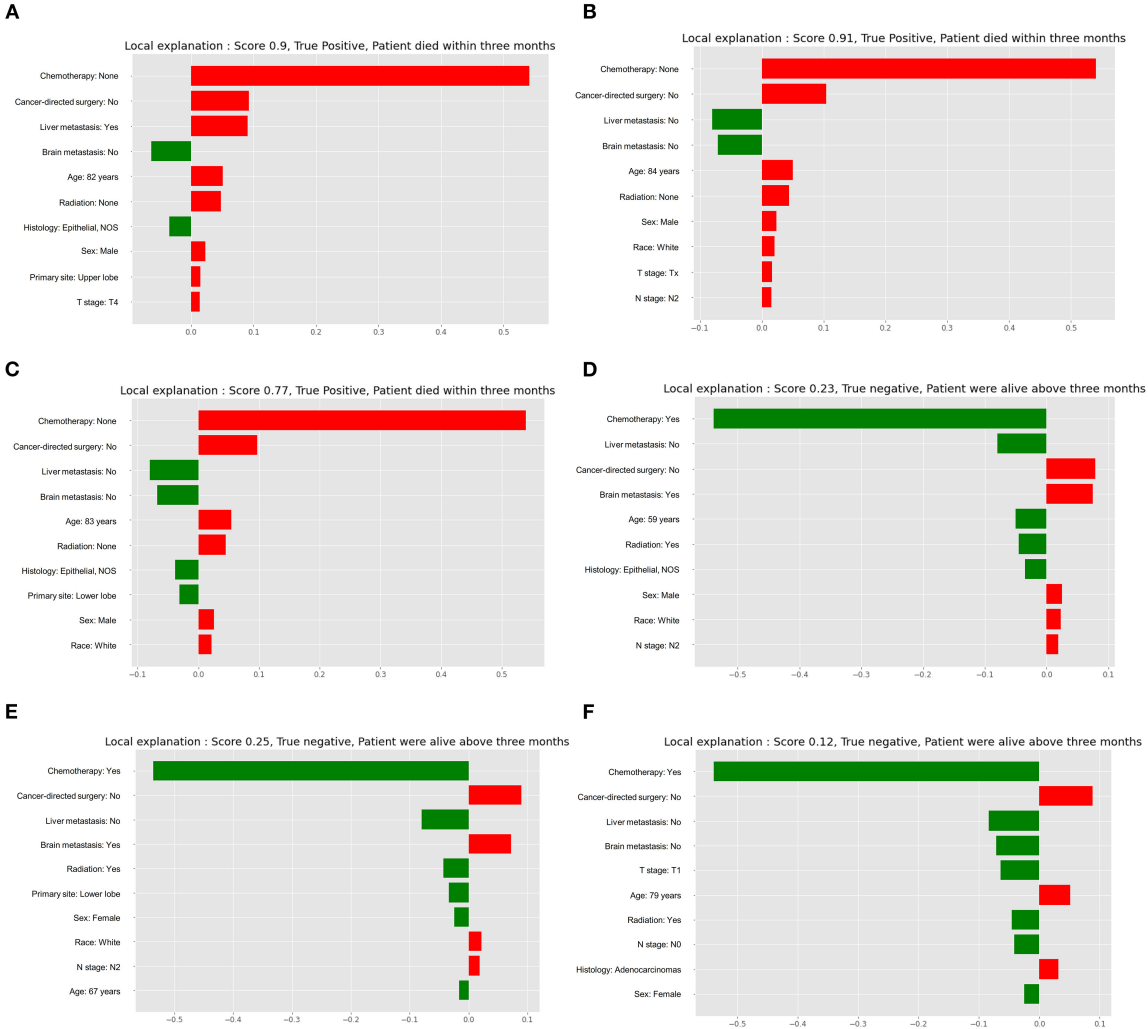
Local interpretable model explainer for six individual cases. **(A)** The first true positive case; **(B)** The second true positive case; **(C)** The third true positive case; **(D)** The first true negative case; **(E)** The second true negative case; **(F)** The third true negative case. Features with a green bar indicates protective prognostic factors, and those with red bar represents contributing risk factors. The x-axis depicts how much each predictor added or subtracted to the final probability for the specific patient.

Explainability of models was conducted to rank the predictors and visualize their contributions to an individual's mortality outcome. The three individuals in Figures 5A–C that died within 3 months had a high anticipated 3 month mortality (True positive). The three cases in Figures 5D–F that were alive longer than 3 months had a low anticipated probability of 3 month mortality (True negative). The top 10 variables were sorted based on the weight of each variable that was presented by the length of the red or green boxes. The red boxes indicated risk factors for 3 month mortality, and the green boxes indicated protective factors for 3 month mortality. In the first case, it depicted a specific individual who had a 90.0% chance of dying within 3 months. In this case, no chemotherapy, no cancer-directed surgery, liver metastasis, an age of 82 years, radiation, male, upper lobe, and T4 stage were all risk contributors to 3 month mortality, while no brain metastasis and epithelial (NOS) were protective factors for the outcome. The top three important predictors were chemotherapy, cancer-directed surgery, and liver metastasis, and the weight of each predictor could be obtained by referring to the X-axis.

### Importance of model predictors

Predictor importance evaluation was performed using H_2_O automatic machine learning in the entire cohort of patients. The learning curve demonstrated that the H_2_O automatic machine learning was neither underfit nor overfit (Supplementary Figure 4). Chemotherapy, followed by liver metastasis, radiation, and brain metastasis were the top four important contributors to the outcome among all variables in both training (Supplementary Figure 5A) and validation (Supplementary Figure 5B) groups according to SHAP summary plots. SHAP summary plot is able to depict the contribution of the features for each instance (row of data). The Kaplan-Meier survival curves showed great discrimination between patients with and without the treatment of chemotherapy (*P* < 0.0001, log-rank test, Supplementary Figure 5C) or radiation (*P* < 0.0001, log-rank test, Supplementary Figure 5D). When compared to patients without liver or brain metastasis, individuals who had either of these metastases had significantly worse overall survival outcomes (*P* < 0.0001, log-rank test, Supplementary Figures 5E,F). For primary site, histology, race, sex, tumor (T) stage, node (N) stage, and cancer-directed surgery, survival curves were also plotted (Supplementary Figures 6–12). The relationship between age and the outcome was also displayed using the H_2_O automatic machine learning, showing that the 3 month mortality increased over time as age grew (Supplementary Figure 13A). Additionally, when age was divided into deciles, two categorical age groups could be obtained (Supplementary Figure 13B): patients with a relatively high risk of 3 month mortality (above the partial dependence line) and patients with a relatively low risk of 3 month mortality (belove the dependence line).

### Risk stratification

**Figure 6:**
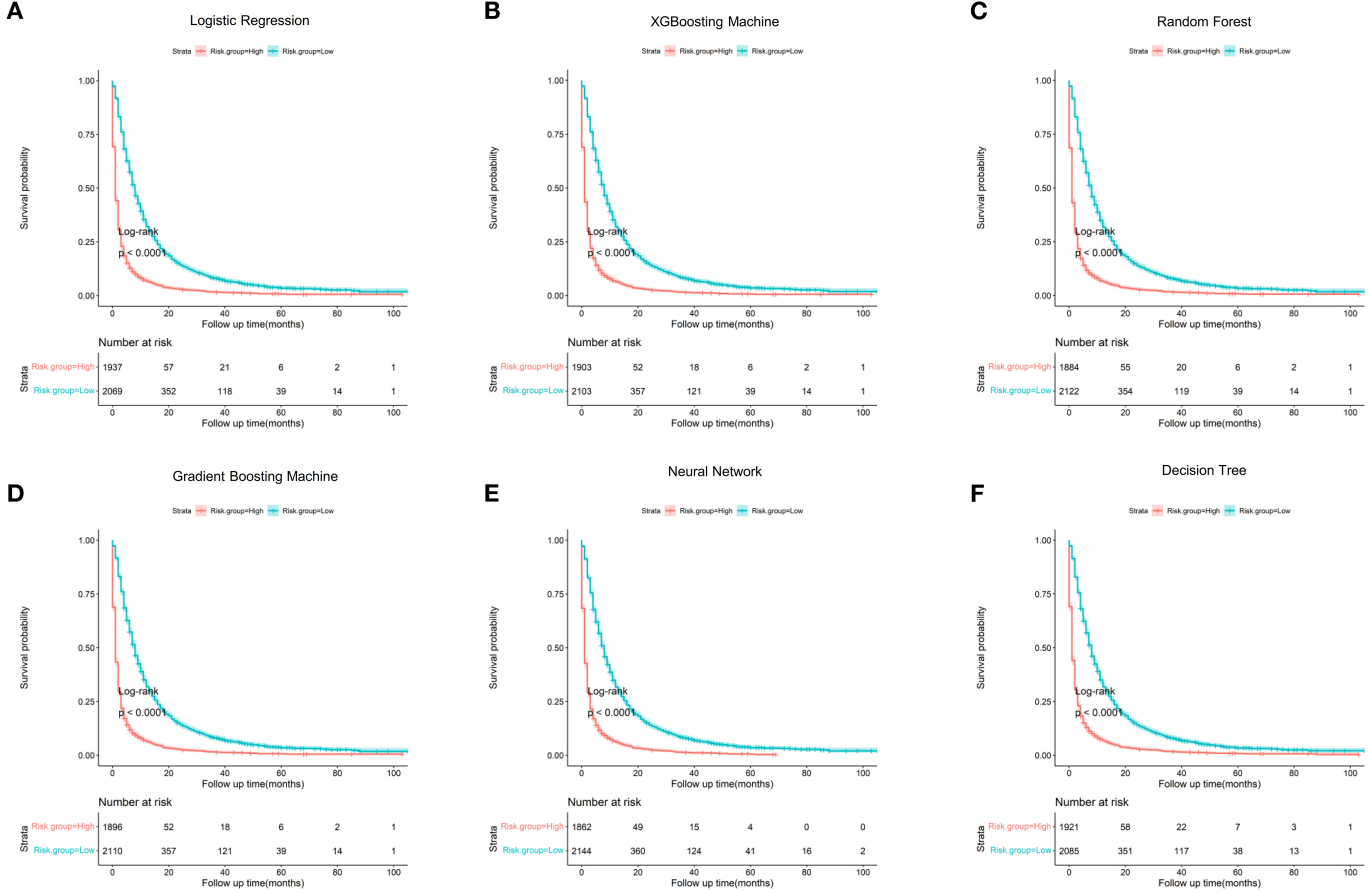
Kaplan-Meier survival curves stratified by risk group (All *P* < 0.001, log-rank test). **(A)** Logistic Regression; **(B)** XGBooting Machine; **(C)** Random Forest; **(D)** Gradient Boosting Machine; **(E)** Neural Network; **(F)** Decision Tree.

Based on the optimal cut-off value (40.00%), patients were categorized into a low-risk group and a high-risk group (Table 4). Patients in the low-risk group had a probability of <40%, and patients in the high-risk group had a probability of >40.00%. Patients in the high-risk group had more than 3 times the odds of dying within 3 months as compared to patients in the high-risk group (*P* < 0.001), with regard to the gradient boosting machine. The six algorithms are shown in Figure 6 with the survival curves stratified by the risk group, and all six algorithms demonstrated a significant difference in survival outcome between the two risk groups (All *P* < 0.001, log-rank test).

**Table 4:** Risk stratification based on the optimal cut-off value in the models.

**Approaches**	**Patients** **(*n* = 4,006)**	**Probability**		***P*-value**
		**Predicted**	**Actual**	
**Logistic regression**				
Low risk (≤ 40.00%)	2,069	22.18%	23.88% (494/2,069)	<0.001
High risk (>40.00%)	1,937	77.19%	77.13% (1,494/1,937)	
**XGBoosting machine**				
Low risk (≤ 40.00%)	2,103	22.16%	23.92% (503/2,103)	<0.001
High risk (>40.00%)	1,903	78.06%	78.03 (1,485/1,903)	
**Random forest**				
Low risk (≤ 40.00%)	2,122	30.10%	24.18% (513/2,122)	<0.001
High risk (>40.00%)	1,884	69.43%	78.29% (1,475/1,884)	
**Gradient boosting machine**				
Low risk (≤ 40.00%)	2,110	22.37%	23.93% (505/2,110)	<0.001
High risk (>40.00%)	1,896	78.04%	78.22% (1,483/1,896)	
**Neural network**				
Low risk (≤ 40.00%)	2,144	19.01%	24.53% (526/2,144)	<0.001
High risk (>40.00%)	1,862	77.36%	78.52% (1,462/1,862)	
**Decision tree**				
Low risk (≤ 40.00%)	2,085	22.14%	24.36% (508/2,085)	<0.001
High risk (>40.00%)	1,921	77.62%	77.04% (1,480/1,921)	

XGBooting, eXtreme Gradient Boosting.

## Discussion

This study developed models using machine learning approaches, and the optimal model is selected after comprehensively assessing and comparing the prediction performance. The gradient boosting machine approach performed best, followed by XGBooting machine approach and logistic regression based on the overall of the 13 prediction measures. The optimal model demonstrated favorable discrimination and calibration, and as a result, the models' explainability was performed in this optimal model to improve the models' trust and transparency. Additionally, the importance of variables was investigated in individuals and among the whole population.

In the entire cohort of patients, the 3 month mortality was up to 48.5% and the median survival time was 4.0 months, both of which were consistent with prior studies (9, 10). Literature reported that the median survival period for lung cancer patients with bone metastases was 4.0 to 5.0 months (9, 10). In the study, we found that the 3 month mortality was significantly correlated with age, primary site, histology, race, sex, T stage, N stage, brain metastasis, liver metastasis, cancer-directed surgery, radiation, and chemotherapy. These variables were easily available, and the majority of these variables had already been proven in previous studies (23–25). To be more specific, older age, male, advanced T or N stage, and brain or live metastasis were risk factors, while adenocarcinoma, cancer-directed surgery, radiation, and chemotherapy were protective factors.

The use of surgical procedures, such as stable fixation of long bones and spine decompressive surgery, to maintain or improve patients' functional outcomes and overall performance status has increased in recent years due to the development of therapeutic modalities (11, 12). But the major concern was whether post-operative recovery or surgery-related complications would be a balance between benefits and harms for patients in their remaining survival time. To address this issue, several survival scoring systems were proposed to optimize the balance between surgical interventions and survival outcomes among patients with bone metastases. In 2021, for instance, Owari et al. (26) proposed a scoring system to predict survival outcomes among patients with bone metastases in a cohort of 489 patients with genitourinary cancers; Katagiri et al. (27) developed a new prognostic scoring system to predict survival outcome in a cohort of 808 patients with skeletal metastases; More recently, Chi et al. (28) created a nomogram to predict survival prognosis among 326 bone metastases patients with head and neck cancer. However, the above scoring systems are derived from several primary cancer types which might have different patterns of metastases, and thus the prognostic ability might cannot appropriately work among bone metastases in particular lung cancer patients who had a relatively very short life expectancy. Kang et al. (29) created nomograms for predicting overall survival, progression-free survival, and time-to-progression at 5 years in 714 patients with early-stage non-small cell lung cancer after stereotactic ablative radiation therapy. The C-indexes for the nomograms of overall survival, progression-free survival, and time-to-progression were 0.72, 0.66, and 0.59, respectively, in the validation cohort. Pruksakorn et al. (10) established a prognostic scoring system to predict life expectancy in a total of 505 lung cancer patients with bone metastases, Rades et al. (30) proposed a scoring system to estimate survival among lung cancer patients after treating with radiotherapy (*n* = 120), and Lei et al. (31) constructed a score to predict survival time among spinal metastatic lung cancer patients (*n* = 73). Although the above scoring systems were designed specifically for lung cancer patients with or without bone metastases, these scoring systems had very limited samples and evaluations of prediction metrics, and thus their generalization needed further investigations.

The present study introduced six approaches to develop corresponding six models and used 13 metrics to assess and compare the prediction performance of these models. Subsequently, the optimal model was selected to present model explainability, and six individual specific cases were illustrated in the study. In these cases, the explainer model ranked and displayed the top 10 predictors according to the relative importance of each predictor individually. The explainer model not only calculated the individually predicted probability of 3 month mortality in each given case, but also enabled users to have a deep understanding of how the model arrived at the conclusion. As a result, users' trusts and model transparency could be remarkably enhanced (32). Predictor importance was also evaluated using H_2_O automatic machine learning in the entire patient cohort, and it demonstrated that the top four important predictors were chemotherapy, radiation, liver metastasis, and brain metastasis. Therefore, taking steps to administer chemotherapy or radiation while preventing liver and brain metastases would significantly improve the prognosis for survival. Additionally, this study sheds a light on the connection between age and 3 month mortality.

Patients were categorized into a low-risk group and a high-risk group in order to undertake therapy strategies individually. Compared to patients in the high-risk group, those in the high-risk group had more than three times the odds of developing ab early death (*P* < 0.001). Patients in the high-risk group were advised to receive radiotherapy alone, the best supportive care, or minimally invasive techniques like cementoplasty, because they might not be able to recover quickly enough to benefit from surgery. Since patients in the low-risk group had a somewhat longer life expectancy and were more likely to benefit from more invasive therapies, aggressive procedures, such as excisional surgery for spine or bone metastases, and long-term radiations, were better carried out on these individuals.

### Limitations

Despite the fact that this study presented and validated more accurate models, it still had some restrictions. For starters, although there might be significant predictors of survival such as the quantity and location of bone metastases, the systemic immune-inflammation index (29), and the Charlson comorbidity index (29), these variables were not available in the SEER database. With the easily available clinical data, our proposed model already had favorable prediction performance based on discrimination and calibration. Furthermore, even though internal validation of the model yielded encouraging findings, external validation of the model was not carried out, necessitating additional research before the model could be generalized.

## Conclusions

Using machine learning techniques, this study offers a number of models, and the optimal model is found after thoroughly assessing and contrasting the prediction performance of each model. The optimal model can be a pragmatic risk prediction tool and is capable of identifying lung cancer patients with bone metastases who are at high risk for 3 month mortality, informing risk counseling, and aiding clinical treatment decision-making. It is better advised for patients in the high-risk group to have radiotherapy alone, the best supportive care, or minimally invasive procedures like cementoplasty.

## Data availability statement

Publicly available datasets were analyzed in this study. This data can be found here: The database can be accessed publicly and provide patient data without specific identification, so ethics approval and informed consent were not required. We obtained approval to access the database of the National Cancer Institute in the United States using the reference number 23489-Nov2020 (https://seer.cancer.gov/). The human data was in accordance with the Declaration of Helsinki.

## Ethics statement

Ethical review and approval was not required for the study on human participants in accordance with the local legislation and institutional requirements. Written informed consent for participation was not required for this study in accordance with the national legislation and the institutional requirements.

## Author contributions

XS and SW oversaw data collection. YC and ML performed the analysis. SW and ML drafted the manuscript. ML has full access to all the data in the study and had final responsibility for the decision to submit for publication. All authors conceived and designed the analysis, provided clinical interpretation of the findings, reviewed, edited, and confirmed their acceptance of the final submitted version.

## Funding

This study was supported by National Orthopedics and Sports Rehabilitation Clinical Medical Research Center Innovation Fund Project (2021-NCRC-CXJJ-PY-20) and Hainan Province Clinical Medical Center.

## Conflict of interest

The authors declare that the research was conducted in the absence of any commercial or financial relationships that could be construed as a potential conflict of interest.

## Publisher's note

All claims expressed in this article are solely those of the authors and do not necessarily represent those of their affiliated organizations, or those of the publisher, the editors and the reviewers. Any product that may be evaluated in this article, or claim that may be made by its manufacturer, is not guaranteed or endorsed by the publisher.

## Supplementary material

Supplementary Figure 1 The receiver operating curve for the six approaches.

Supplementary Figure 2 The box plots of predicted probabilities of 3 month mortality between patients who actually died at or within 3 months (“Yes”) and patients who actually were alive for above 3 months (“No”). **(A)** Logistic Regression; **(B)** XGBooting Machine; **(C)** Random Forest; **(D)** Gradient Boosting Machine; **(E)** Neural Network; **(F)** Decision Tree.

Supplementary Figure 3 Decision curve analysis of the six approaches. The horizontal gray line indicates treated-for-none scheme and another reference line indicates treated-for-all scheme.

Supplementary Figure 4 The learning curve for H_2_O machine learning.

Supplementary Figure 5 Importance of model predictors. **(A)** SHAP summary plot in the training group; **(B)** SHAP summary plot in the validation group; **(C)** Kaplan-Meier survival curve stratified by chemotherapy (*P* < 0.0001, log-rank test); **(D)** Kaplan-Meier survival curve stratified by radiation (*P* < 0.0001, log-rank test); **(E)** Kaplan-Meier survival curve stratified by liver metastasis (*P* < 0.0001, log-rank test); **(F)** Kaplan-Meier survival curve stratified by brain metastasis (*P* < 0.0001, log-rank test). In the SHAP summary plot, the y-axis is the features and the x-axis is the Shapley value in each case. Color represents the eigenvalue (red indicates high and blue indicates low) and enables us to match how the change of eigenvalue affects risk. The overlapping points jitter in the direction of y-axis, it can enable us to understand how the Shapley value distribute in each feature, and these features are sorted according to their importance.

Supplementary Figure 6 Kaplan-Meier survival curve stratified by histology (*P* < 0.0001, log-rank test).

Supplementary Figure 7 Kaplan-Meier survival curve stratified by N stage (*P* < 0.0001, log-rank test).

Supplementary Figure 8 Kaplan-Meier survival curve stratified by primary site (*P* < 0.0001, log-rank test).

Supplementary Figure 9 Kaplan-Meier survival curve stratified by race (*P* < 0.0001, log-rank test).

Supplementary Figure 10 Kaplan-Meier survival curve stratified by sex (*P* < 0.0001, log-rank test).

Supplementary Figure 11 Kaplan-Meier survival curve stratified by caner-directed surgery (*P* < 0.0001, log-rank test).

Supplementary Figure 12 Kaplan-Meier survival curve stratified by T stage (*P* < 0.0001, log-rank test).

Supplementary Figure 13 The association between age and response (3 month mortality) evaluated using the H_2_O machine learning. **(A)** Partial dependence plot for age; **(B)** Individual conditional expectation for gradient boosting machine according to decile.

Supplementary Table 1 Code and description about cancer-directed surgery.

Supplementary Table 2 Code and description about radiation.

Supplementary Table 3 Code and description about chemotherapy.

Supplementary Table 4 A comparison of clinical characteristics among patients with and without surgery. ^a^Indicates continuity adjusted Chi-Square; ^b^Indicates American Indian/AK Native, Asian/Pacific Islander. SD, standard deviation; NOS, not otherwise specified; T stage, tumor stage; N stage, node stage.

Supplementary Table 5 Approaches and parameters.
