# Supplementary figures and images for: Machine learning approaches for prediction of early death among lung cancer patients with bone metastases using routine clinical characteristics: An analysis of 19,887 patients
Source: Front Public Health. 2022 Oct 6;10:1019168. doi: 10.3389/fpubh.2022.1019168 (PMC9583680; doi:10.3389/fpubh.2022.1019168)

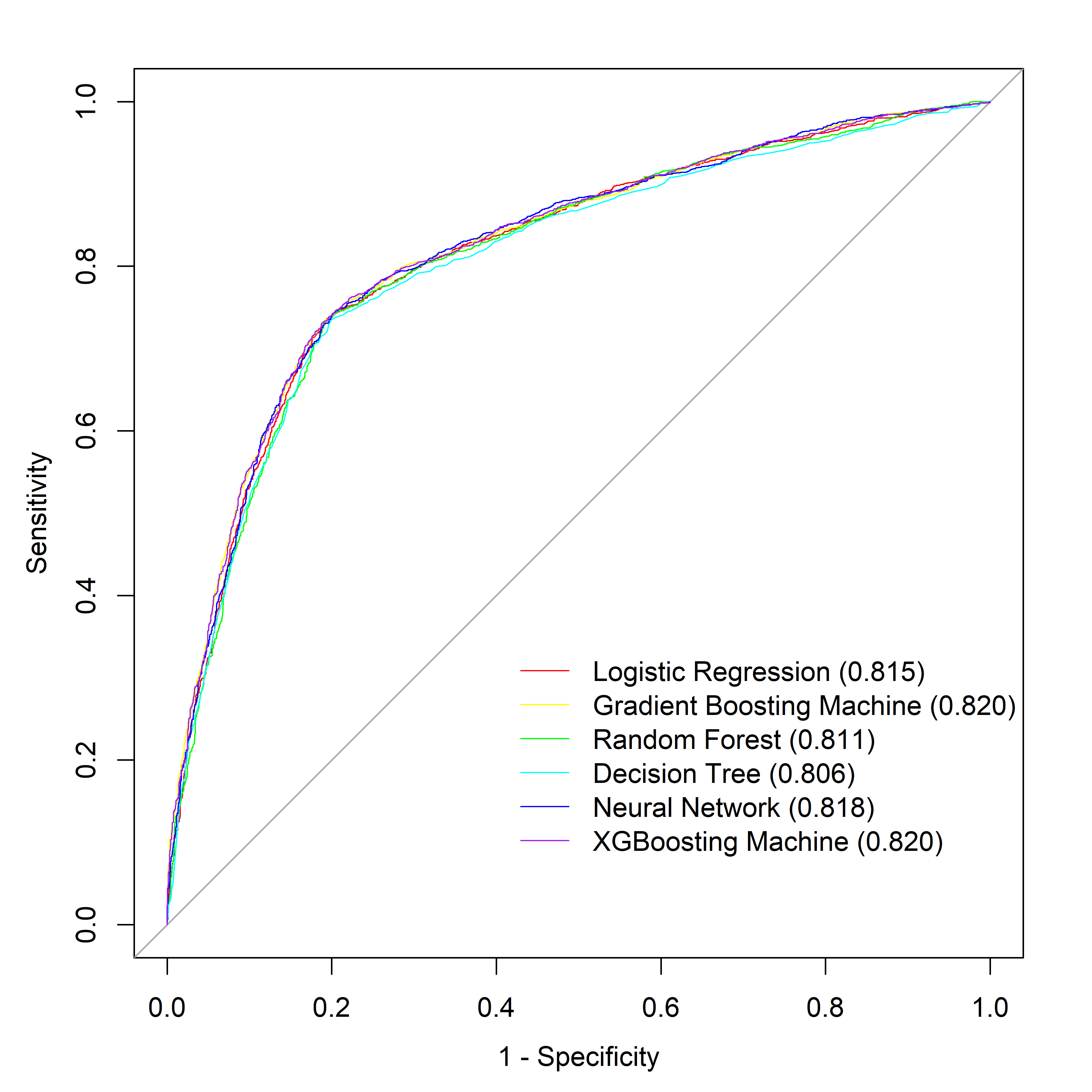

Supplement: Supplementary Figure 1 — The receiver operating curve for the six approaches. [file Image_1.TIF]

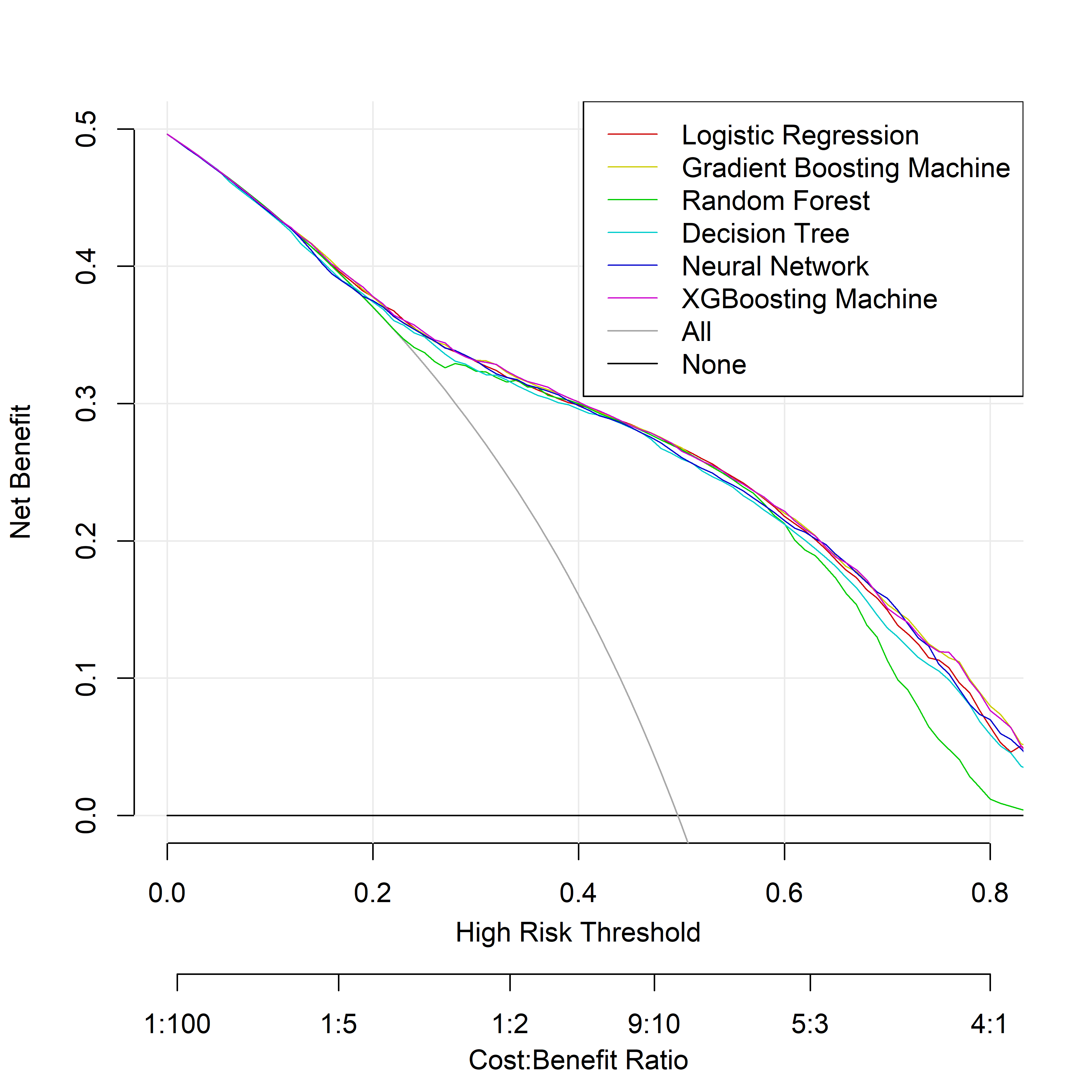

Supplement: Supplementary Figure 3 — Decision curve analysis of the six approaches. The horizontal gray line indicates treated-for-none scheme and another reference line indicates treated-for-all scheme. [file Image_3.TIF]

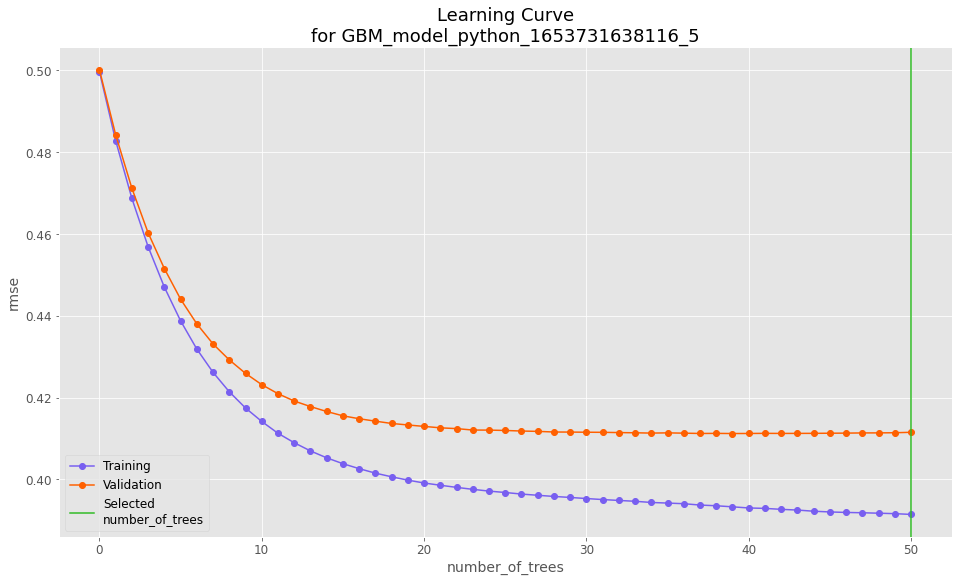

Supplement: Supplementary Figure 4 — The learning curve for H2O machine learning. [file Image_4.TIF]

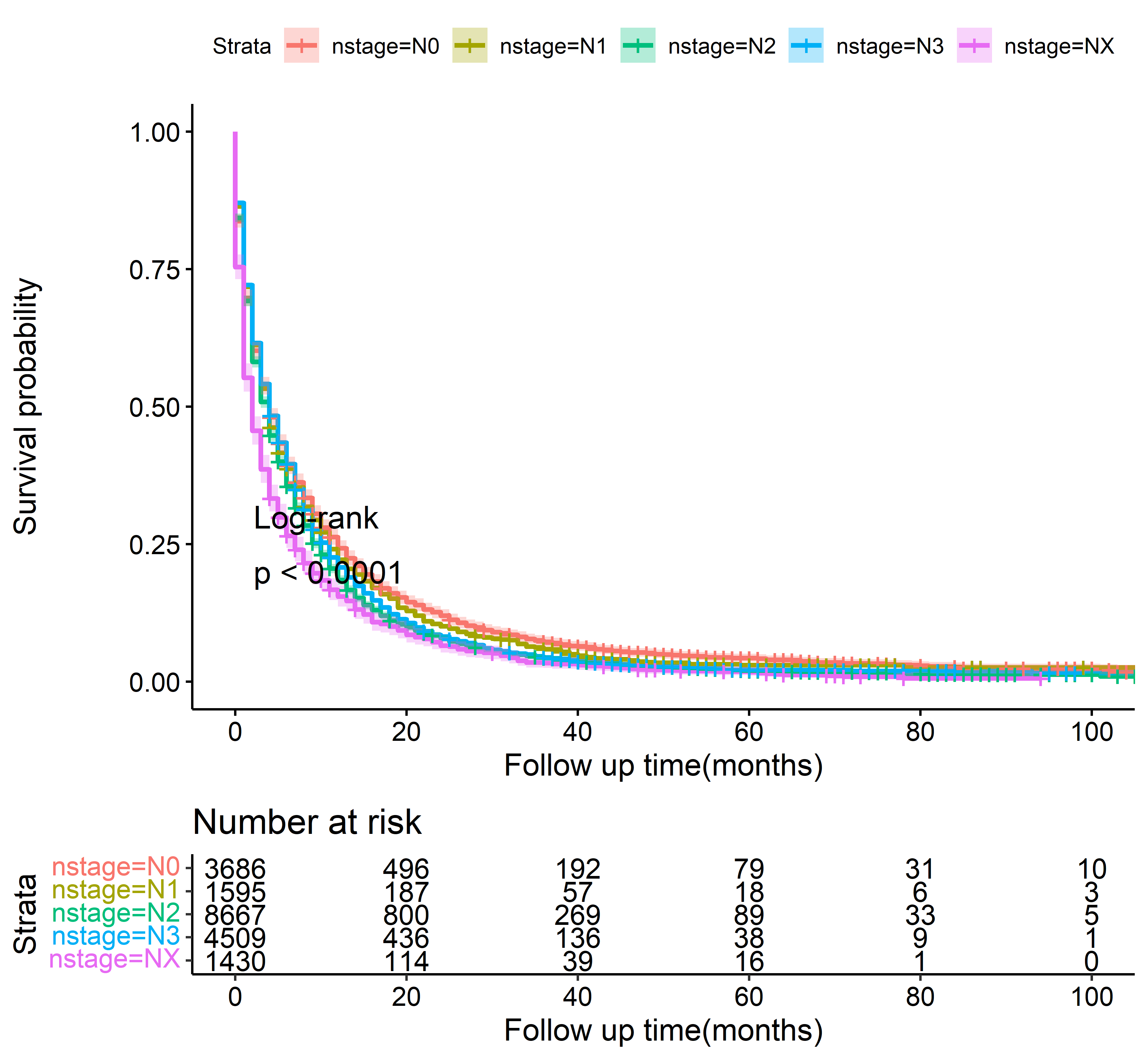

Supplement: Supplementary Figure 6 — Kaplan-Meier survival curve stratified by histology (P < 0.0001, log-rank test). [file Image_6.TIF]

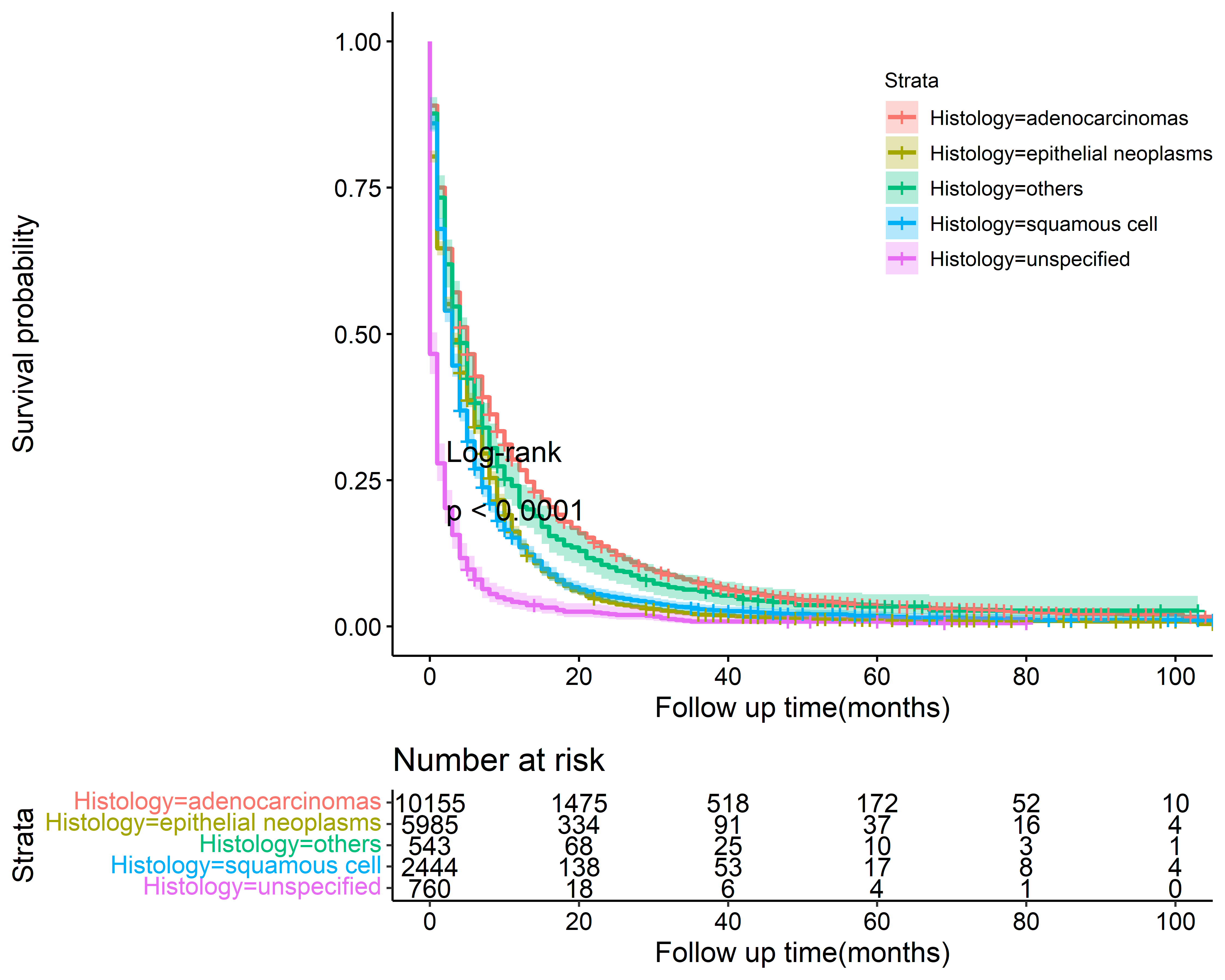

Supplement: Supplementary Figure 7 — Kaplan-Meier survival curve stratified by N stage (P < 0.0001, log-rank test). [file Image_7.TIF]

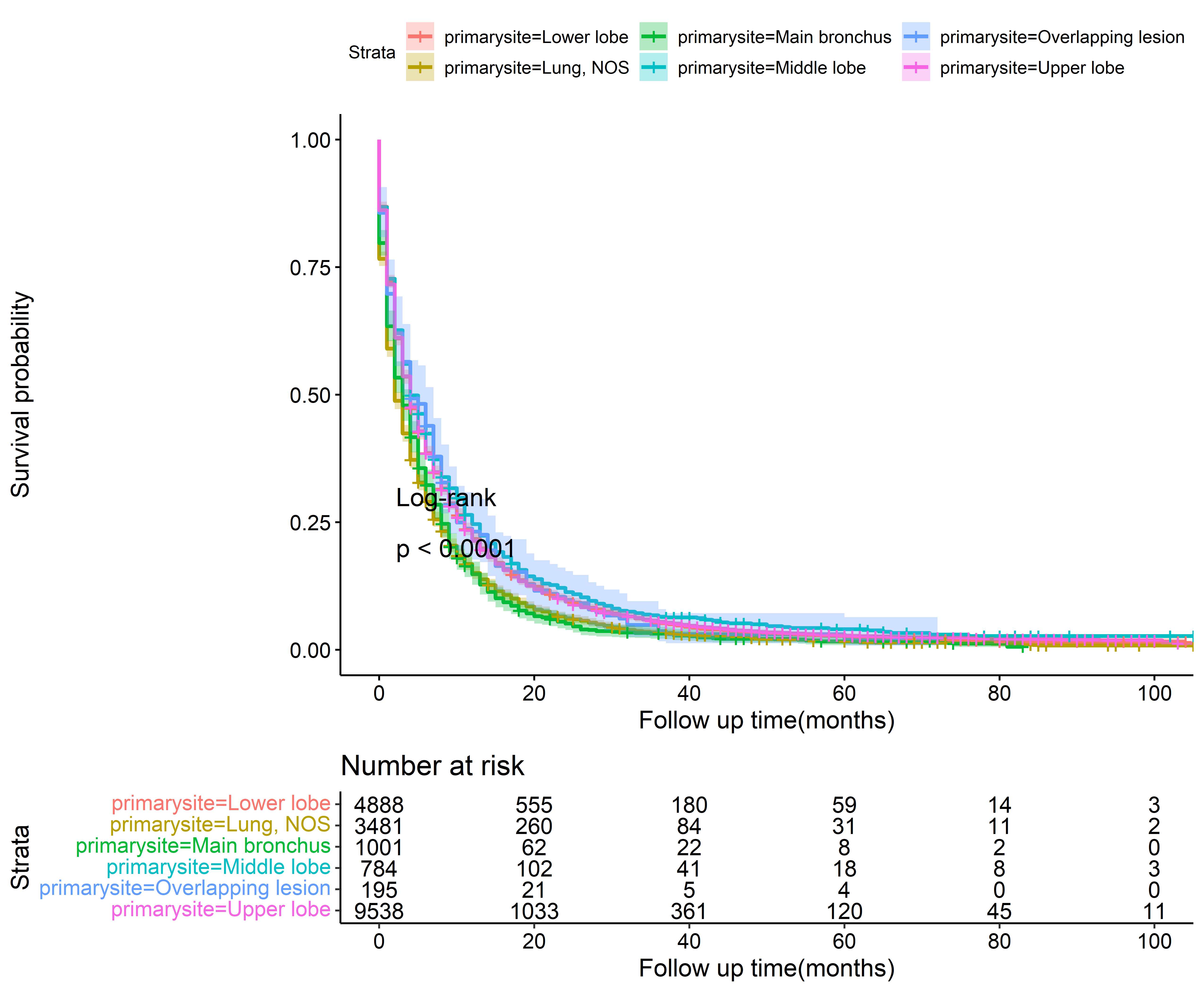

Supplement: Supplementary Figure 8 — Kaplan-Meier survival curve stratified by primary site (P < 0.0001, log-rank test). [file Image_8.TIF]

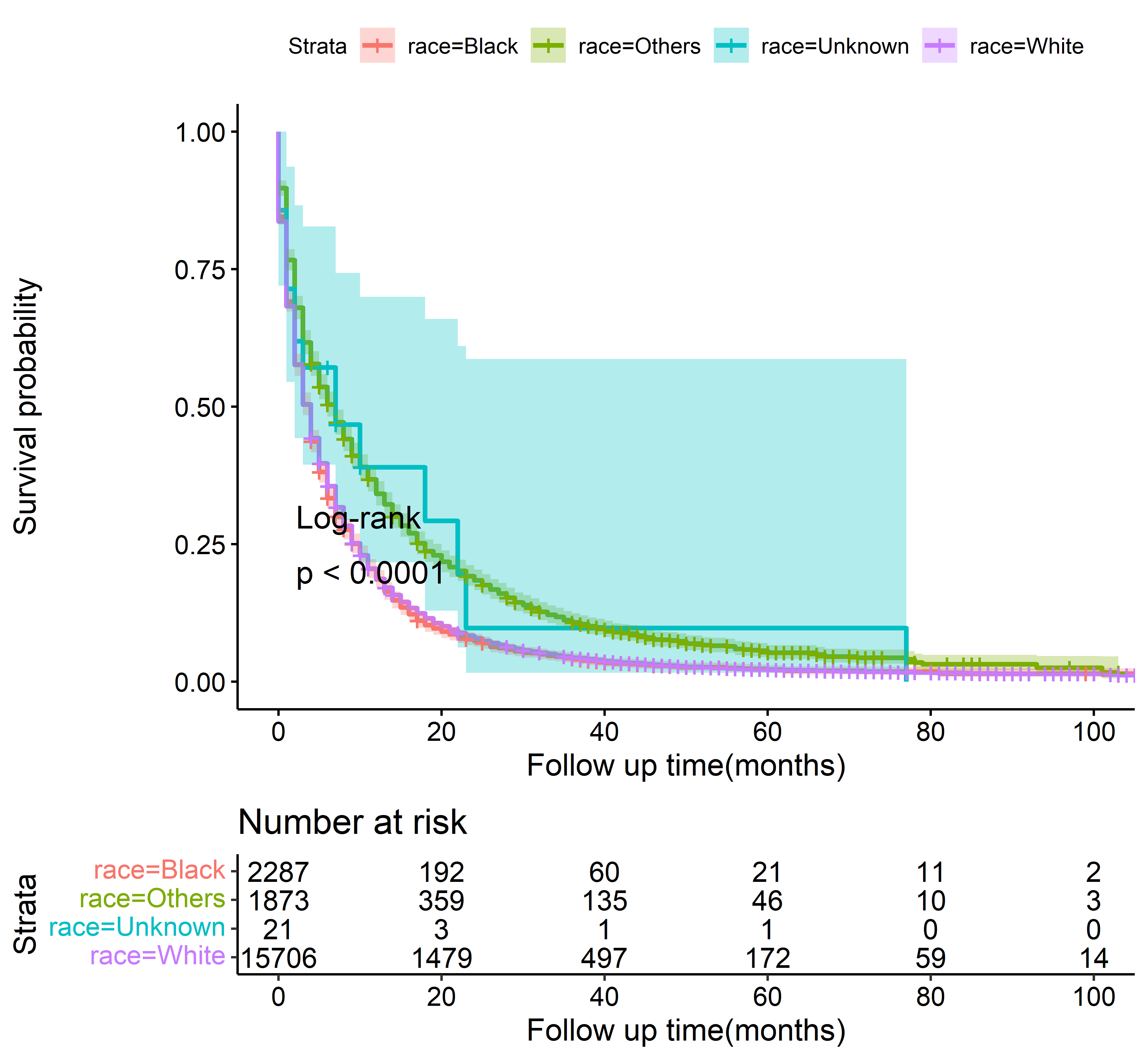

Supplement: Supplementary Figure 9 — Kaplan-Meier survival curve stratified by race (P < 0.0001, log-rank test). [file Image_9.TIF]

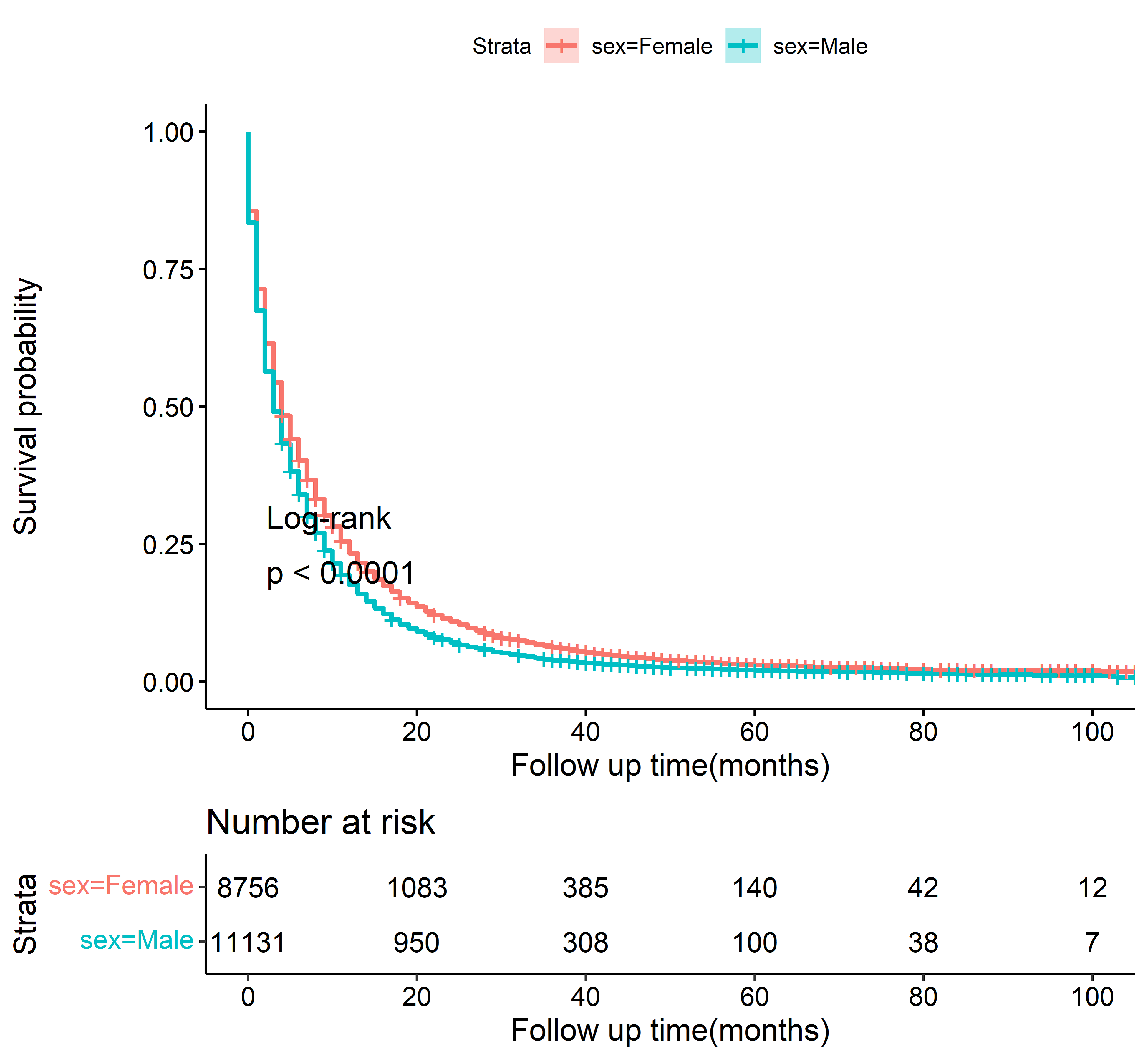

Supplement: Supplementary Figure 10 — Kaplan-Meier survival curve stratified by sex (P < 0.0001, log-rank test). [file Image_10.TIF]

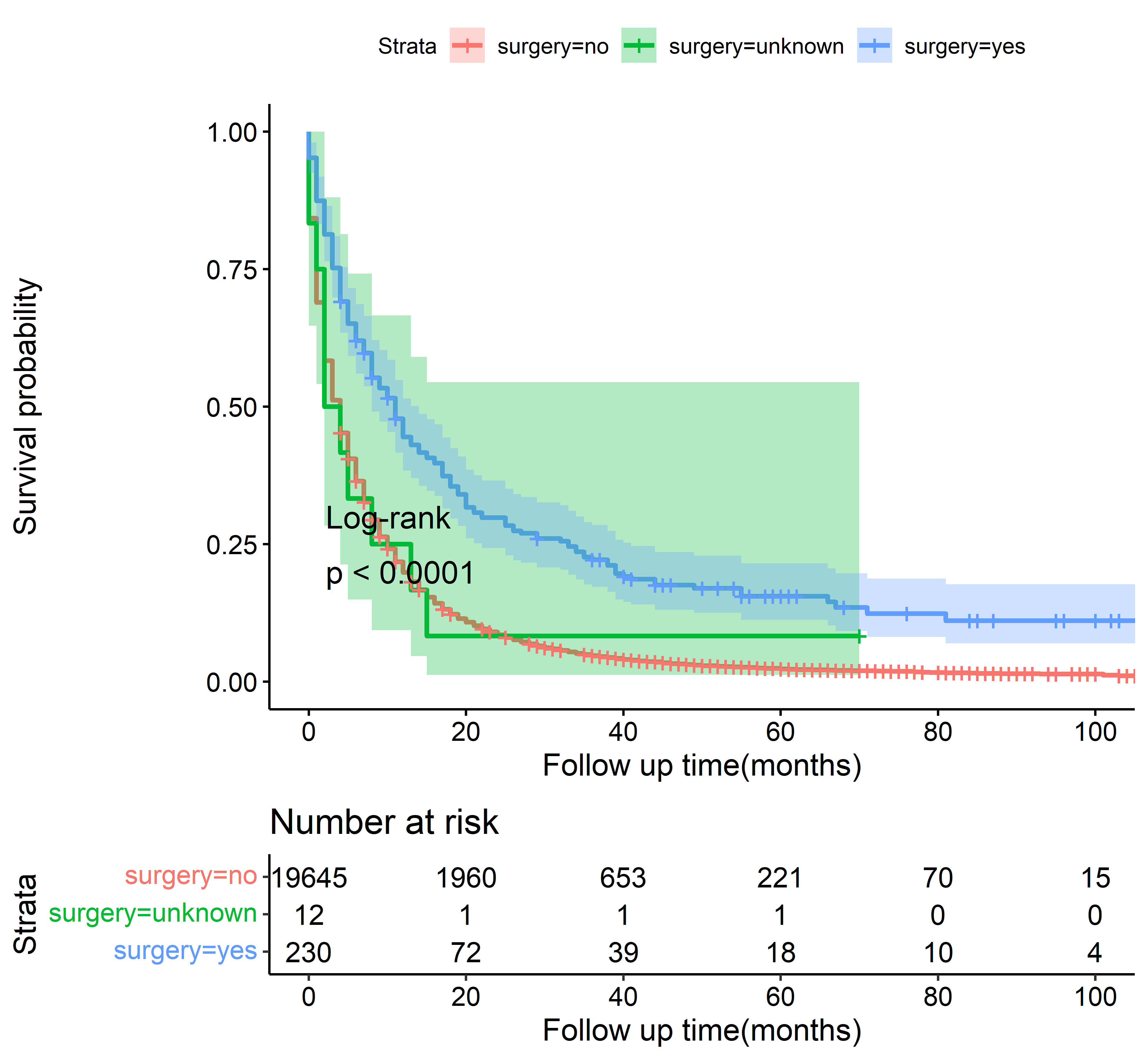

Supplement: Supplementary Figure 11 — Kaplan-Meier survival curve stratified by caner-directed surgery (P < 0.0001, log-rank test). [file Image_11.TIF]

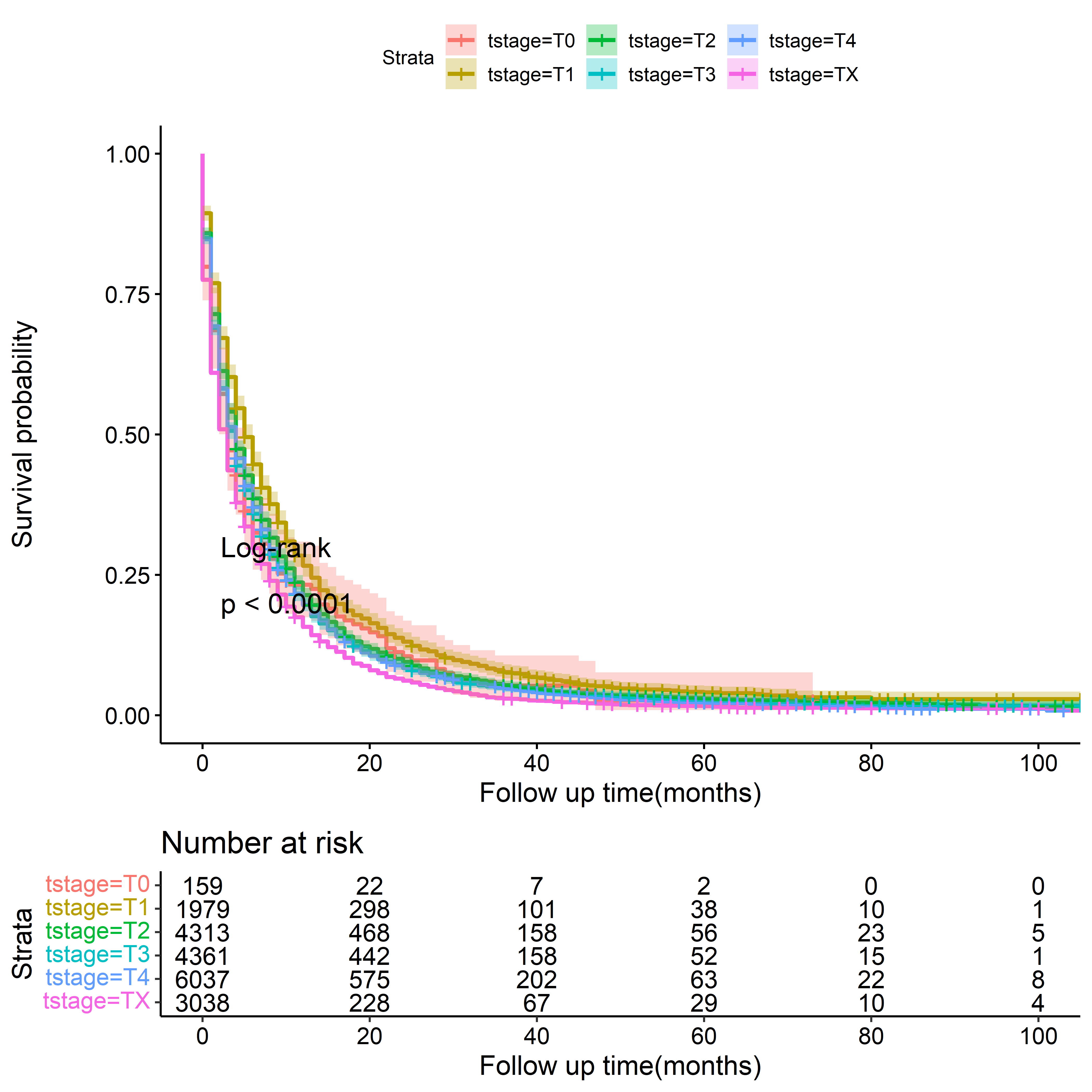

Supplement: Supplementary Figure 12 — Kaplan-Meier survival curve stratified by T stage (P < 0.0001, log-rank test). [file Image_12.TIF]
