# Supplementary material for: Machine learning approaches for prediction of early death among lung cancer patients with bone metastases using routine clinical characteristics: An analysis of 19,887 patients
Source: Front Public Health. 2022 Oct 6;10:1019168. doi: 10.3389/fpubh.2022.1019168 (PMC9583680; doi:10.3389/fpubh.2022.1019168)
Supplement: Supplementary Table 1 — Code and description about cancer-directed surgery. [file Data_Sheet_1.docx]

| **Supplementary table 1.** Code and description about cancer-directed surgery. | | |
| --- | --- | --- |
| Code | | Description |
| Surgery | | |
| 0 | Surgery performed | |
| No surgery | | |
| 1 | Surgery not recommended | |
| 2 | Contraindicated due to other conditions; Autopsy Only case (1973-2002) | |
| 5 | Patient died before recommended surgery | |
| 6 | Unknown reason for no surgery | |
| 7 | Patient or patient’s guardian refused | |
| Unknown if surgery performed | | |
| 8 | Recommended, unknown if done | |
| 9 | Unknown if surgery performed; Death Certificate Only case; Autopsy only case (2003+) | |
| 14 | Blank | |

| **Supplementary table 2**. Code and description about radiation. | |
| --- | --- |
| Code | Description |
| None/unknown |  |
| 0 | None/Unknown; diagnosed at autopsy |
| Radiation performed | |
| 1 | Beam radiation |
| 2 | Radioactive implants |
| 3 | Radioisotopes |
| 4 | Combination of 1 with 2 or 3 |
| 5 | Radiation, NOS—method or source not specified |
| 6 | Other radiation (1973-1987 cases only) |
| None/unknown |  |
| 7 | Patient or patient's guardian refused radiation therapy |
| 8 | Radiation recommended, unknown if administered |

| **Supplementary table 3**. Code and description about chemotherapy. | |
| --- | --- |
| Code | Description |
| 0 | None/Unknown |
| 1 | Yes |

| **Supplementary table 4.** A comparison of clinical characteristics among patients with and without surgery. | | | | | |
| --- | --- | --- | --- | --- | --- |
| Characteristics | Overall | Surgery | | | P ^a^ |
|  |  | No | Unknown | Yes |  |
| n | 19887 | 19645 | 12 | 230 |  |
| Age (mean (SD)) | 68.46 (11.05) | 68.51 (11.03) | 67.33 (10.40) | 64.05 (11.46) | <0.001 |
| Primary site (%) |  |  |  |  | <0.001 |
| Main bronchus | 1001 (5.0) | 988 (5.0) | 0 (0.0) | 13 (5.7) |  |
| Upper lobe | 9538 (48.0) | 9434 (48.0) | 2 (16.7) | 102 (44.3) |  |
| Middle lobe | 784 (3.9) | 771 (3.9) | 0 (0.0) | 13 (5.7) |  |
| Lower lobe | 4888 (24.6) | 4810 (24.5) | 2 (16.7) | 76 (33.0) |  |
| Overlapping lesion | 195 (1.0) | 191 (1.0) | 0 (0.0) | 4 (1.7) |  |
| Lung, NOS | 3481 (17.5) | 3451 (17.6) | 8 (66.7) | 22 (9.6) |  |
| Histology (%) |  |  |  |  | <0.001 |
| Unspecified neoplasms | 760 (3.8) | 756 (3.8) | 2 (16.7) | 2 (0.9) |  |
| Epithelial neoplasms, NOS | 5985 (30.1) | 5953 (30.3) | 3 (25.0) | 29 (12.6) |  |
| Squamous cell neoplasms | 2444 (12.3) | 2409 (12.3) | 1 (8.3) | 34 (14.8) |  |
| Adenomas and adenocarcinomas | 10155 (51.1) | 10007 (50.9) | 6 (50.0) | 142 (61.7) |  |
| Others | 543 (2.7) | 520 (2.6) | 0 (0.0) | 23 (10.0) |  |
| Race (%) |  |  |  |  | 0.026 |
| Black | 2287 (11.5) | 2247 (11.4) | 2 (16.7) | 38 (16.5) |  |
| Others ^b^ | 1873 (9.4) | 1856 (9.4) | 3 (25.0) | 14 (6.1) |  |
| Unknown | 21 (0.1) | 20 (0.1) | 0 (0.0) | 1 (0.4) |  |
| White | 15706 (79.0) | 15522 (79.0) | 7 (58.3) | 177 (77.0) |  |
| T stage (%) |  |  |  |  | <0.001 |
| T0 | 159 (0.8) | 159 (0.8) | 0 (0.0) | 0 (0.0) |  |
| T1 | 1979 (10.0) | 1949 (9.9) | 0 (0.0) | 30 (13.0) |  |
| T2 | 4313 (21.7) | 4255 (21.7) | 0 (0.0) | 58 (25.2) |  |
| T3 | 4361 (21.9) | 4305 (21.9) | 0 (0.0) | 56 (24.3) |  |
| T4 | 6037 (30.4) | 5967 (30.4) | 0 (0.0) | 70 (30.4) |  |
| TX | 3038 (15.3) | 3010 (15.3) | 12 (100.0) | 16 (7.0) |  |
| N stage (%) |  |  |  |  | <0.001 |
| N0 | 3686 (18.5) | 3603 (18.3) | 1 (8.3) | 82 (35.7) |  |
| N1 | 1595 (8.0) | 1570 (8.0) | 1 (8.3) | 24 (10.4) |  |
| N2 | 8667 (43.6) | 8582 (43.7) | 0 (0.0) | 85 (37.0) |  |
| N3 | 4509 (22.7) | 4479 (22.8) | 1 (8.3) | 29 (12.6) |  |
| NX | 1430 (7.2) | 1411 (7.2) | 9 (75.0) | 10 (4.3) |  |
| Brain metastasis (%) |  |  |  |  | <0.001 |
| No | 14599 (73.4) | 14417 (73.4) | 3 (25.0) | 179 (77.8) |  |
| Unknown | 635 (3.2) | 622 (3.2) | 6 (50.0) | 7 (3.0) |  |
| Yes | 4653 (23.4) | 4606 (23.4) | 3 (25.0) | 44 (19.1) |  |
| Liver metastasis (%) |  |  |  |  | <0.001 |
| No | 13240 (66.6) | 13049 (66.4) | 2 (16.7) | 189 (82.2) |  |
| Unknown | 589 (3.0) | 579 (2.9) | 6 (50.0) | 4 (1.7) |  |
| Yes | 6058 (30.5) | 6017 (30.6) | 4 (33.3) | 37 (16.1) |  |
| Radiation (No/Yes, %) | 9859/10028 (49.6/50.4) | 9733/9912 (49.5/50.5) | 12/0 (100.0/0.0) | 114/116 (49.6/50.4) | 0.002 |
| Chemotherapy (No/Yes, %) | 9035/10852 (45.4/54.6) | 8933/10712 (45.5/54.5) | 11/1 (91.7/8.3) | 91/139 (39.6/60.4) | 0.001 |
| Early death (No/Yes, %) | 10232/9655 (51.5/48.5) | 10053/9592 (51.2/48.8) | 6/6 (50.0/50.0) | 173/57 (75.2/24.8) | <0.001 |
| **Notes:** ^a^ indicates continuity adjusted Chi-Square; ^b^ indicates American Indian/AK Native, Asian/Pacific Islander.  **Abbreviations:** SD, standard deviation; NOS, not otherwise specified; T stage, tumor stage; N stage, node stage. | | | | | |

| **Supplementary table 5**. Approaches and parameters. | |
| --- | --- |
| **Approaches** | **Parameters** |
| Logistic regression | C=0.1, random_state=42 |
| XGBoosting machines | base_score=0.5, booster='gbtree', colsample_bylevel=1, colsample_bynode=1, colsample_bytree=1, enable_categorical=False, gamma=0, gpu_id=-1, importance_type=None, interaction_constraints='', learning_rate=0.125, max_delta_step=0, max_depth=3, min_child_weight=7, missing=nan, monotone_constraints='()', n_estimators=108, n_jobs=8, num_parallel_tree=1, predictor='auto', random_state=42, reg_alpha=0, reg_lambda=1, scale_pos_weight=1, subsample=1, tree_method='exact', use_label_encoder=False, validate_parameters=1, verbosity=None. |
| Random forests | max_depth=4, max_features='log2', min_samples_leaf=53, min_samples_split=63, n_estimators=49, random_state=42. |
| Gradient boosting machines | max_features='auto', min_samples_leaf=4, min_samples_split=8, random_state=42. |
| Decision tree | max_depth=39, max_features='log2', min_samples_leaf=42, min_samples_split=29, random_state=42. |
| Neural network | random_state=42,activation='relu',alpha=0.0001,batch_size='auto',beta_1=0.9, beta_2=0.999, early_stopping=False,epsilon=1e-08,hidden_layer_sizes=(100), learning_rate='constant', learning_rate_init=0.001,max_iter=200, momentum=0.9, n_iter_no_change=10, nesterovs_momentum=True, power_t=0.5,shuffle=True, tol=0.0001, validation_fraction=0.1,verbose=False, warm_start=False |
